# Molecular phenotyping of oxidative stress in diabetes mellitus with point-of-care NMR system

**DOI:** 10.1038/s41514-020-00049-0

**Published:** 2020-10-05

**Authors:** Weng Kung Peng, Lan Chen, Bernhard O. Boehm, Jongyoon Han, Tze Ping Loh

**Affiliations:** 1 Precision Medicine–Engineering Group, International Iberian Nanotechnology Laboratory, Braga, Portugal; 2 BioSystems & Micromechanics IRG (BioSyM), Singapore-MIT Alliance for Research and Technology (SMART) Centre, Singapore, Singapore; 3 Lee Kong Chian School of Medicine, Nanyang Technological University, Singapore, Singapore; 4 Ulm University Medical Centre, Department of Internal Medicine 1, Ulm University, Ulm, Germany; 5 Imperial College London, London, UK; 6 Department of Electrical Engineering and Computer Science, Massachusetts Institute of Technology, 36-841, 77 Massachusetts Avenue, Cambridge, MA 02139 USA; 7 Department of Biological Engineering, Massachusetts Institute of Technology, 36-841, 77 Massachusetts Avenue, Cambridge, MA 02139 USA; 8 Department of Laboratory Medicine, National University Hospital, 5 Lower Kent Ridge Road, Singapore, 119074 Singapore

**Keywords:** Metabolic disorders, Pathogenesis, Biological techniques

## Abstract

Diabetes mellitus is one of the fastest-growing health burdens globally. Oxidative stress, which has been implicated in the pathogenesis of diabetes complication (e.g., cardiovascular event), remains poorly understood. We report a new approach to rapidly manipulate and evaluate the redox states of blood using a point-of-care NMR system. Various redox states of the hemoglobin were mapped out using the newly proposed (pseudo) two-dimensional map known as *T*_1_–*T*_2_ magnetic state diagram. We exploit the fact that oxidative stress changes the subtle molecular motion of water proton in the blood, and thus inducing a measurable shift in magnetic resonance relaxation properties. We demonstrated the clinical utilities of this technique to rapidly stratify diabetes subjects based on their oxidative status in conjunction to the traditional glycemic level to improve the patient stratification and thus the overall outcome of clinical diabetes care and management.

## Introduction

Diabetes mellitus (DM) is one of the fastest-growing health burdens that is projected to affect 592 million people worldwide by 2035^1^. DM is defined by a persistent elevation of plasma glucose concentration. Under chronic hyperglycemic condition, glucose is nonenzymatically attached to protein (glycation), which has deleterious effects on its structure and function. Hence, glycated hemoglobin A_1c_ (HbA_1c_), which reflects the overall glycemic burden of an individual over the previous 2–3 months, is now used to diagnose the disease^2^. It is the recommended biomarker for monitoring long-term glucose control of DM patients, and for risk stratification^3–5^.

However, HbA_1c_ does not adequately reflect all the pathology associated with DM. In particular, restoring HbA_1c_ level to near-normal level does not necessarily translate into a significant reduction of the cardiovascular event, a diabetes complication commonly associated with oxidative stress^6^. In addition, subjects with stable chronic hyperglycemia due to glucokinase mutations were found to have unexpectedly lower prevalence of micro-/macrovascular complication^7,8^. A major pathological effect of diabetes mellitus is the chronic oxidative–nitrosative stress, and the recently reported carbonyl^9^ and methylglyoxal stress^10^, which drives many of the secondary complications of diabetes including nephropathy, retinopathy, neuropathy, and cardiovascular diseases^11^. Oxidative–nitrosative stress can damage nucleic acids, lipids, and proteins, which severely compromise the cellular health and induce a range of cellular responses, leading ultimately to cell death^12–14^. Direct measurement of oxidative stress and susceptibility in patients may improve the prediction of disease-associated risks related to oxidative stress, and hence the long-term diabetes care and management program^15,16^.

Currently, an individual’s oxidative status cannot be easily characterized in detail using routinely available biomarkers in clinical practice and/or at point-of-care (PoC)^17^. This has impeded the understanding of the pathological effects of acute and prolonged exposure to oxidative stress. The reactive oxygen species (ROS) and reactive nitrogen species (RNS), which are often reactive and short-lived, may disrupt the redox state of biological tissues/cells (e.g., red blood cells (RBCs), plasma). Several methods have been developed to detect the redox properties of the blood using the optical^16,18^ or magnetic properties^19,20^ of the inorganic iron-chelate of hemoglobin (Hb) and plasma albumin.

Electron spin resonance is commonly used to directly detect the ROS/RNS^21,22^. However, the approach is hampered by inherent sample stability issues and limited sensitivity^23^. Stable molecular products formed from reactions with ROS/RNS, such as the oxidation targets (e.g., lipid, protein, nucleic acid) are measurable using a range of spectrophotometric and mass spectrometry (MS) assays^21^. Nevertheless, fluorescent-staining often causes cell-toxicity^24,25^, and therefore these assays may not provide information that reflects in vivo conditions. Ultraviolet-visible light spectroscopy has poor spectral resolution and limited sensitivity. Furthermore, globin-associated free radical in Hb is not optically visible^26^ (Supplementary Figs. 1–3). MS-based analysis of ROS/RNS reaction products is a powerful and sensitive technique that reveals detailed chemistry of these species, yet requires substantial sample preparation and therefore difficult to be deployed as a rapid screening tool^27^. The advantages and disadvantages of each technology were summarized (Supplementary Table 1).

**Fig. 1:**
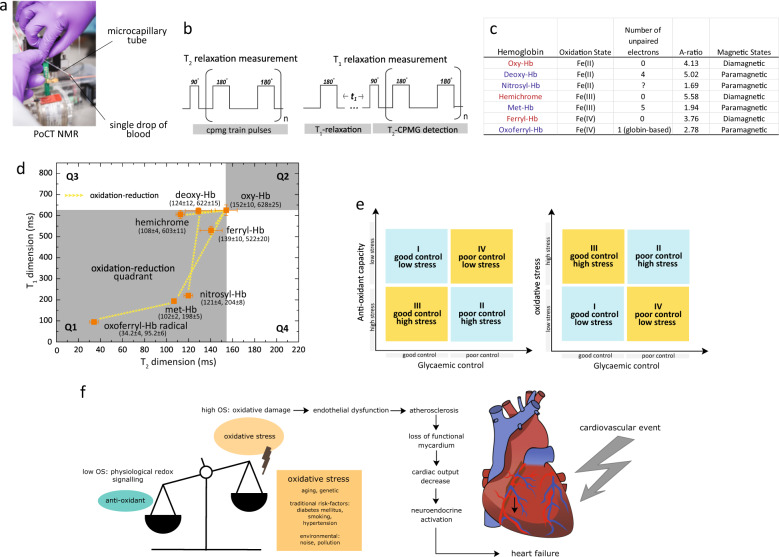
Functional sub-phenotyping of oxidative stress with micro MR analysis approach. **a** The developed bench-top sized micro MR system consists of a commercial console, detection circuit coil mounted on a micro stage, and a palm-sized 0.5 T permanent magnet. The microcapillary tube which contains a single drop of blood is slotted into the radio-frequency probe for micro MR analysis. The read-out completes in <5 min. **b** The rf pulse sequences used were standard CPMG pulse sequence and standard inversion-recovery experiment (with CPMG detection) for the *T*_2_ relaxations, and *T*_1_ relaxations measurements, respectively. In order to obtain high signal-to-noise ratio under a relatively inhomogeneous magnetic environment, an array of echoes (a few thousands) within a very short echo interval (in the order of μs) were used to acquire spin-echoes from <4 μL sample volume of packed RBCs or plasma. **c** Redox reaction of the iron-heme in various oxidation states: Fe^2+^, Fe^3+^, Fe^4+^, and globin-radical Fe^4+^, which were chemically induced in in vitro environment (Methods Online). The hemoglobins were in two possible magnetic states: diamagnetic (red) and paramagnetic state (blue). **d** Various redox states of hemoglobin mapped out using the proposed *T*_1_–*T*_2_ magnetic state diagram. The coordinates (in ms) were oxy-Hb (*T*_2_ = 152 ± 10, *T*_1_ = 628 ± 25), deoxy-Hb (*T*_2_ = 124 ± 12, *T*_1_ = 622 ± 15), met-Hb (*T*_2_ = 102 ± 2, *T*_1_ = 198 ± 5), ferryl Hb (*T*_2_ = 139 ± 10, *T*_1_ = 522 ± 20), oxo ferryl-Hb (*T*_2_ = 34.2 ± 4, *T*_1_ = 95.2 ± 6), nitrosyl-Hb (*T*_2_ = 121 ± 4, *T*_1_ = 204 ± 8), and hemichrome (*T*_2_ = 108 ± 4, *T*_1_ = 603 ± 11). Three different samplings were taken from the same donor, and the results were reported as mean ± standard error measurement. The data is represented by box-plot format. **e** A quadrant chart of diabetic subject stratified into subgroups based on their oxidative status (e.g., antioxidant capacity (left) and oxidative stress (right), in which the oxidative stress here includes nitrosative stress and peroxidative stress), in association with their glycemic index (e.g., HbA_1c_). **f** Relationship between elevation of oxidative stress and development of DM-related complications (e.g., cardiovascular event).

We herein report an approach to rapidly quantify the composite redox state of the Hb/plasma^28,29^ by direct measurement of proton relaxation rates of (predominantly) bulk water using a bench-top sized micro magnetic resonance relaxometry (micro MR) system^29–33^. The non-destructive nature of the micro MR analysis allows oxidative stress to be artificially introduced in ex vivo environment using different biochemical compounds (e.g., nitrite, peroxide) in a controlled manner (Fig. 1a–c). This allows functional assessment of the oxidative susceptibility, tolerance, and capacity of a given sample. This yields significantly richer and clinically useful information about the oxidative stress levels of the blood within an individual, as compared to routine biomarkers. To enumerate the various redox states of the Hb (e.g., Fe^2+^, Fe^3+^, Fe^4+^, and globin-associated radical Fe^4+^) and the plasma, two-dimensional relaxations map, known as *T*_1_–*T*_2_ magnetic state diagram was proposed (Fig. 1d)^34,35^. This magnetic state diagram allows visualization and identification of the intermediate redox states and the transient, dynamic pathways of the blood sample.

MR relaxometry is a technique that measures relaxation rate, by acquiring spin-echoes of (predominantly) water content of the cells/tissues using conventional nuclear magnetic resonance (NMR) spectroscopy and magnetic resonance imaging (MRI) system. Recent advances in NMR system miniaturization have raised the prospect of applying these techniques in point-of-care diagnostic setting^36–38^. These applications include immuno-magnetic labeling based detection (e.g., tumor cells^39–41^, tuberculosis^42^, and magneto-DNA detection of bacteria^43^) and label-free micro MR detection of various diseases (e.g., oxygenation^44^/oxidation^31^ level of the blood, malaria screening^30,33,45^, diabetes mellitus^28^, and hemoglobinopathies^35^).

We applied micro MR analysis on whole blood samples to stratify diabetic subjects into subgroups based on their oxidative status levels in association with their glycemic index (Fig. 1e). Assessment of oxidative status by measuring the redox state of whole blood was shown to be highly time- and patient-specific, revealing information that is potentially critical for clinical diagnostic, monitoring, and prognostic purposes (Fig. 1f).

## Results

### *T*_1_–*T*_2_ magnetic state diagram

Redox homeostasis is a fundamental biological process, which maintains the balance between ambient antioxidant and pro-oxidant activities. The red blood cell is an important biological agent in ameliorating oxidative stress^46,47^. On the other hand, free heme is one of the major sources of redox-active iron, which has deleterious effect on DNA/protein and RBCs themselves. The fundamental process of oxidative (and nitrosative) stress involves the process of electron transfer, which leads to eventual formation of oxidized products. The oxidized product is much more stable and measurable using proton NMR relaxometry.

Here, we chemically induced (Methods Online) and characterized various redox states of the red blood cell and represented them using *T*_1_–*T*_2_ magnetic resonance relaxation state diagram (Fig. 1d). Each Hb species has specific oxidation states (e.g., Fe^2+^, Fe^3+^, Fe^4+^, globin-associated radical of Fe^4+^ or its’ corresponding complexes) that are bound to specific neighboring proteins and dissipate energy via unique relaxations mechanism in both the longitudinal (*T*_1_) and transverse (*T*_2_) relaxation frames. The *T*_2_ and *T*_1_ relaxation times measurement were performed using the standard Carr–Purcell–Meiboom–Gill (CPMG) pulse sequence^48^ and inversion recovery observed by CPMG, respectively. The pairing of both relaxation times forms a specific *T*_1_–*T*_2_ relaxometry coordinate, which is unique to each redox state.

These relaxation times reflect predominantly of proton nuclei from the bulk water, which came into contact with macromolecular protein (e.g., hemoglobin, albumin)^49^. Water is an attractive natural molecular network probing system as it forms hydrogen bonds with practically all other macromolecules (e.g., protein) that are present in human circulation^49,50^. Therefore, a subtle change of the molecular environment can induce a measurable change in the proton relaxation rate. Early works demonstrating proton relaxation rate is dependent on the blood oxygenation level had been carried out by Thulborn et al.^51^ and Gomori et al.^52^. These discoveries were eventually applied to measure brain activity known as functional MRI^53^.

Oxyhemoglobin (oxy-Hb) which has the lowest reduced ferrous (Fe^2+^) state is the predominant Hb species in circulation. The oxy-Hb has been provisionally assigned to the center of the state diagram, which has four quadrants (i.e., Q1). Due to the semi-solid structure of RBC and oxidation process which reduces the proton relaxation time, the redox pathways of RBC were mapped out predominantly into Q1 (Fig. 1d).

Electrons in the *d* sub-orbital of iron hemoglobin can exist in various paired or unpaired conditions, rendering them into two possible magnetic states, i.e., diamagnetic and paramagnetic states, respectively. Hb with at least one unpaired electron, i.e., deoxygenated hemoglobin (deoxy-Hb), methemoglobin (met-Hb), nitrosyl hemoglobin (nitrosyl-Hb), and oxoferryl radical exhibit the effect of paramagnetism with much larger bulk magnetic susceptibility than its’ diamagnetic counterparts i.e., oxy-Hb, ferryl Hb, and hemichrome (HC) (Fig. 1c). The magnetic relativity contributed by paramagnetic ion is highly dependent on its spin state and is directly proportional to *S*(*S* + 1), where *S* is the spin quantum number of the total electron spin^54^. Each of the Hb oxidation states has a unique normalized relaxation constant (A-ratio = *T*_1_/*T*_2_ in Fig. 1c).

### Nitrite-induced ferrous oxidation

**Fig. 2:**
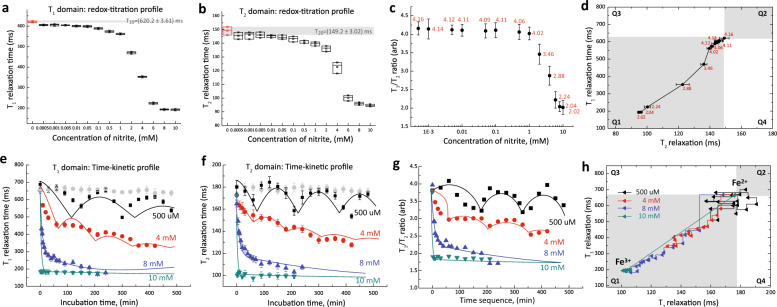
Nitrite-induced ferrous oxidation. Redox-titration profile of red blood cells as function of nitrite concentration in **a** *T*_1_ relaxation and **b** *T*_2_ relaxation domain. The incubation times were 10 min. The control baseline readings were (*T*_2o_ = 149.5, *T*_1o_ = 621.3) ms, which is the readings for oxy-Hb without any nitrite exposure. The limit of detection (LOD) is about 0.0005 mM of nitrite concentration (gray bar). The data is represented by box-plot format. The corresponding concentration-dependent **c** A-ratio, and **d** *T*_1_–*T*_2_ trajectories of the gradual inversion of Fe^2+^ subpopulation to complete formation of Fe^3+^ population. Time-dependent kinetic profile of ferrous oxidation using nitrite concentrations (500 μM, 4 mM, 8 mM, and 10 mM) in **e** *T*_1_ relaxation and **f** *T*_2_ relaxation domain. The corresponding **g** A-ratio, and **h** *T*_1_–*T*_2_ trajectories in the magnetic state diagram. Three different samplings were taken from the same donor, and the results were reported as mean ± standard error measurement. For curve fitting, a general function of exp(−Ct)|cos(Ct)| can be used to describe the oscillatory behavior, where C is concentration.

Freshly collected whole blood samples containing predominantly the oxygenated Hb were oxidized into met-Hb by chemically induced with sodium nitrite (Methods Online). The oxidation levels generated in vitro were independently verified using spectrophotometry (Supplementary Fig. 1a). Redox-titration profile showed a strong dose-dependent curve, where both *T*_1_ and *T*_2_ relaxation times reduced gradually as progressively higher proportion of RBCs were oxidized and increased the volume paramagnetic susceptibility, when the nitrite concentrations were increased from 50 nM to 10 mM (Fig. 2a, b). As the blood sample transited to a complete paramagnetic state (*T*_2_ = 92.8 ms, *T*_1_ = 190.0 ms) from the initial diamagnetic states (*T*_2_ = 149.0 ms, *T*_1_ = 620.0 ms), the A-ratio dropped from 4.16 to 2.02 (*R*^2^ > 0.95, Fig. 2c). As the volume paramagnetic susceptibility increased, this causes the *T*_1_–*T*_2_ trajectory to move downward in Q1 (Fig. 2d).

The dose-dependent reaction was lost when an excess of nitrite (>10 mM) was introduced. This suggested that the oxidant concentration had exceeded antioxidant capacity and all the oxidation states were saturated. At much lower concentration (<100 µM), there was little or no change in the bulk magnetic state of the RBCs, as the majority of the RBCs were able to restore to their original reduced state. Interestingly, a steep transitional oxidation zone was observed within a very narrow range of nitrite concentration; from 1 to 8 mM, which reflected the redox homeostatic responses within the concentration where the cells were biological viable despite the higher than usual chemical concentration above physiological condition. This was informative to the understanding of the functioning of RBCs at cellular and subpopulation levels (Fig. 2a–c).

Further evidence of redox homeostasis was observed in time-dependent kinetic profiles (Fig. 2e, f) over a range of nitrite concentrations (500 µM, 4 mM, 8 mM, and 10 mM). In general, the measured *T*_1_ and *T*_2_ readings changed in an oscillatory manner over time. This may suggest an active mechanism to regulate cellular redox homeostasis. As the RBCs aged, antioxidant capacity is reduced, thereby forming a subpopulation of cell with disproportionately low antioxidant capacity^1^.

The amplitudes of the oscillation decreased as the nitrite concentration was increased from 500 µM to 4 mM (Fig. 2g). At much higher nitrite concentration (>10 mM), the reaction curve decayed rapidly in an exponential manner with an increasingly dampened oscillation. Similar observations were recorded using spectrophotometry (Supplementary Fig. 1). Interestingly, the corresponding kinetic profiles followed an identical path over time in the *T*_1_–*T*_2_ trajectories as the nitrite concentration was increased (Fig. 2h). The oxidation process drove all the trajectories toward a common coordinate (*T*_2_ = 92.8 ms, *T*_1_ = 190.0 ms), where all the RBCs were converted fully into met-Hb. For low nitrite concentration (e.g., 500 μM), however, the *T*_1_–*T*_2_ trajectory circulated around the origin and did not reach the eventual met-Hb coordinates.

### Study design: clinical study with subjects of DM

A cross-sectional study was carried out to stratify DM subjects based on their oxidative status. DM subjects (*n* = 185) who had HbA_1c_ measured in the outpatient clinic as part of their clinical care were included in this study. These subjects had HbA_1c_ ranging from 4 to 16% and the subjects were classified into good glycemic control (<7.0% HbA_1c_) and poor glycemic control (>8.0% HbA_1c_) subgroups^2^. Healthy young male subjects (*n* = 32; age range of 21–40 years, fasting glucose below 5.6 mmol/L, average HbA_1c_ of 5.16 (±0.32)%, and body mass index below 23.5 kg/m^2^) were separately recruited as control subjects. The collected whole blood in EDTA-anticoagulated tubes was centrifuged (14,000 *×* *g*, 5 min) to separate the RBCs and plasma. The micro MR analysis was performed blindly on the freshly collected blood samples or otherwise kept at 4 ˚C within 2 h. Other clinical laboratory tests (e.g., HbA_1c_) were performed in parallel.

### Baseline study: oxidative status of glycated Hb in RBCs

**Fig. 3:**
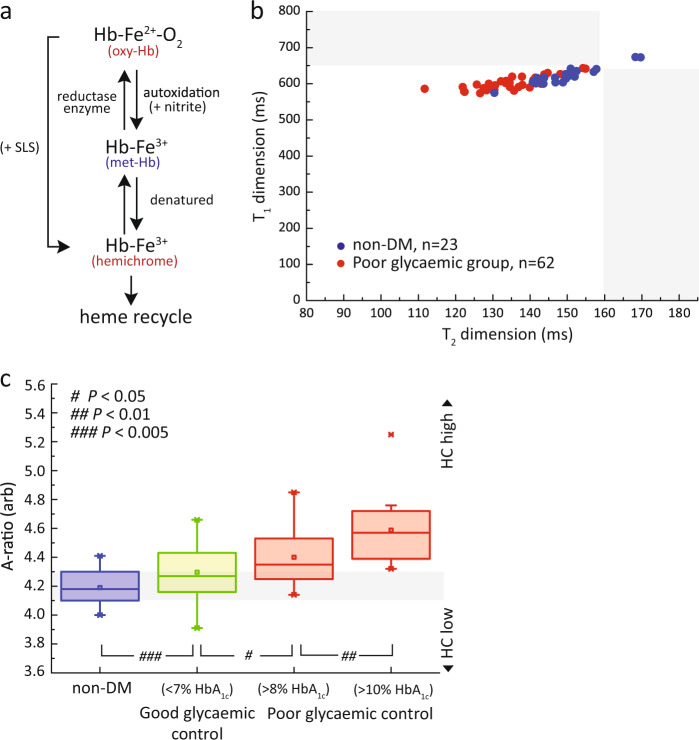
Baseline measurements of oxidative stress in RBCs. **a** In vivo redox oxidation produces denatured Hb, known as the low-spin hemichrome (HC). HC can be chemically induced using sodium salicylate (SLS) in in vitro environment. **b** The *T*_1_–*T*_2_ relaxometry coordinates of RBCs baseline readings of non-DM subjects (blue, *n* = 23) and subjects with poor glycaemic control (red, *n* = 68). **c** Baseline readings of RBCs samples with A-ratio index of subjects with poor glycaemic controls (*n* = 62) and good glycaemic control (*n* = 50) subgroup as compared to healthy non-DM subjects (*n* = 20). The subjects with poor glycaemic controls were further subdivided into >8% HbA_1c_ (*n* = 47) and >10% HbA_1c_ (*n* = 15) subgroups. The A-ratio for the subgroups were in increasing trends for non-DM to DM due to increasing formation of HC (Supplementary Fig. 4). The statistical significance was calculated using the Student’s T-test (two-tailed, unequal variance). The data is represented by box-plot format.

The baseline oxidative status of intact RBCs was measured and mapped using in *T*_1_–*T*_2_ magnetic state diagram. It is observed that DM subjects, in particular, the poor glycemic control (red) had much shorter *T*_1_ and *T*_2_ readings in comparison to non-DM subjects (blue) (Fig. 3a, b). This is highlighted in the A-ratio plot that yielded significantly better resolution (Fig. 3c). The main reason for relaxations reduction was due to the accumulation of denatured Hb (i.e., low-spin hemichrome (HC)) in DM subjects (Fig. 3a). In particular, the HC concentration was markedly elevated in DM subjects with more than 10% HbA_1c_ (Fig. 3c). The origin and effect of HC formation were investigated in vitro (chemically induced) and found to be consistent with the in vivo observation (Supplementary Fig. 4)^26,55^.

### Nitrosative stress test on glycated Hb in RBCs

To further evaluate the ability of RBCs to tolerate the nitrosative stress, we artificially challenged the RBCs with strong oxidant. We established a new protocol to evaluate the optimal concentration of oxidant (Supplementary Figs. 5–7). The concentration range must reflect the homeostatic viable range where the ability to discern the subject-to-subject variability is the largest (highest resolution). Freshly collected RBCs were incubated with 6-mM sodium nitrite for 10 min, washed three times to stop the reaction, and finally resuspended in 1x PBS for micro MR analysis (Methods Online). It worth noting, however, in order to shorten the incubation time, the concentration of the stressors used (i.e., nitrite, H_2_O_2_) was applied at a supra-physiological concentration (in the range of 500–2500-fold) which may exceed and cofound the biological responses or capacity of the cells to counteract the challenge.

**Fig. 4:**
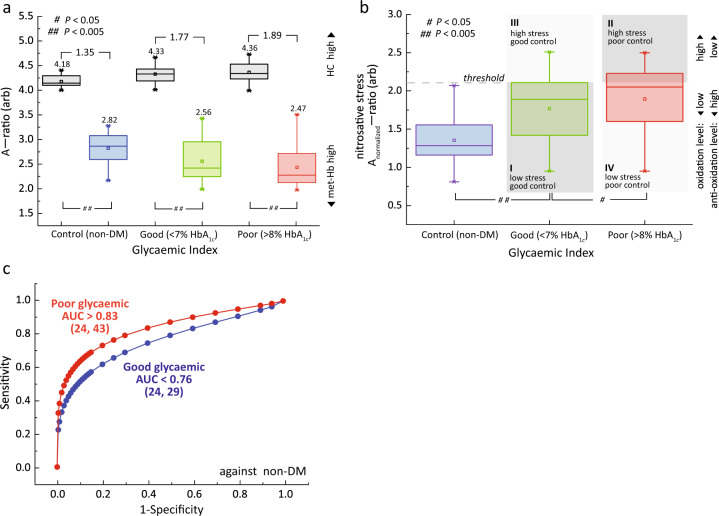
Nitrosative stress test on glycated Hb in RBCs. **a** The A-ratio plot for RBCs in baseline (black) and pretreated sodium nitrite (red, green, blue) for subjects with non-DM subjects (*n* = 24), and DM subjects with their glycemic index in good glycemic control (*n* = 29), and (iii) poor glycemic control (*n* = 43) subgroups. The means and changes in each subgroups were indicated above the box-plots. The data is represented by box-plot format. **b** A quadrant chart of diabetic subjects stratified into subgroups based on their normalized oxidative status (nitrosative stress) in association with their glycemic levels (e.g., HbA_1c_). The threshold of A_nitrosative_-ratio was at 75th percentile that of DM subjects with good glycemic control, and comes with the flexibility of adjusting based on clinical needs (e.g., population genetics). Note that the Y-axis (nitrosative stress) is analogous with Y-axis (peroxidative stress) in the quadrant shown in Fig. 6b. The details of quadrant representations are shown in Fig. 1e. The statistical significance was calculated using the Student’s T-test (two-tailed, unequal variance). The data is represented by box-plot format. **c** The diagnostic accuracies were evaluated using receiver operating characteristic (ROC) for non-DM subjects against DM subjects with good glycemic control (blue) and poor glycemic control (red) subgroups. The number of subjects (*n*) is indicated in parentheses.

Non-DM (*n* = 24) and DM subjects with poor glycemic control (*n* = 43) and good glycemic control (*n* = 29) subgroups were assessed before (black) and after nitrosative stress (colored) (Fig. 4a). The A-ratio for baseline RBCs for non-DM and DM subjects (black) were found to be in increasing trend (due to increasing HC formation), while the nitrosative stress test (colored) resulted in the A-ratio in decreasing trend (due to increasing the amount of met-Hb formation). These results were consistent with the in vivo observation (Fig. 3) and the in vitro stress test conducted (Fig. 2), respectively.

The non-DM subjects had the highest antioxidant (or anti-nitrosative) capacity as compared to DM subjects (*P* > 0.005), while poor glycemic control subgroup has the lowest tolerance to nitrosative stress (Fig. 4b). The actual amount of nitrosative stress (‘normalized’) were calculated by subtracting the background baseline (black, in Fig. 4a). As a result of increased glycation, HbA_1C_ is less stable and prone to oxidation^56,57^ and expectedly the poor glycemic control subgroup (AUC > 0.83) has much higher diagnostic accuracy as compared to good glycemic control subgroup (AUC < 0.76) (Fig. 4c). Significantly, the spread for DM subjects was relatively large (as compared to the non-DM controls) which suggests a large phenotypic variation (e.g., subject-to-subject variability in nitrosative susceptibility), despite being in the same glycemic subgroups (Fig. 4b).

The DM subjects were further sub-stratified into smaller subgroups based on the four quadrants (i.e., Q1–Q4), using the dual independent markers, i.e., the oxidative stress marker (in this case the nitrosative stress) proposed in this study and in conjunction with the traditional glycemic control index (e.g., HbA_1c_). This approach is able to isolate a minority small subgroup in Q3 (subgroup III) consists of subjects with good glycemic control and unexpectedly (high) nitrosative stress. This dispels the conventional belief that every subject in good glycemic control subgroup was presumably healthier (e.g., lower oxidative stress) than poor glycemic control subjects and thereby gave a false-indication about their risk factors on developing diabetes complications. Isolation of subgroup III subjects (early detection) would help to streamline clinical subjects for therapeutic purposes in a timely manner, which demonstrates the closing gap between translational science and clinical medicine.

### Baseline study: glycation and glycoxidation of plasma

**Fig. 5:**
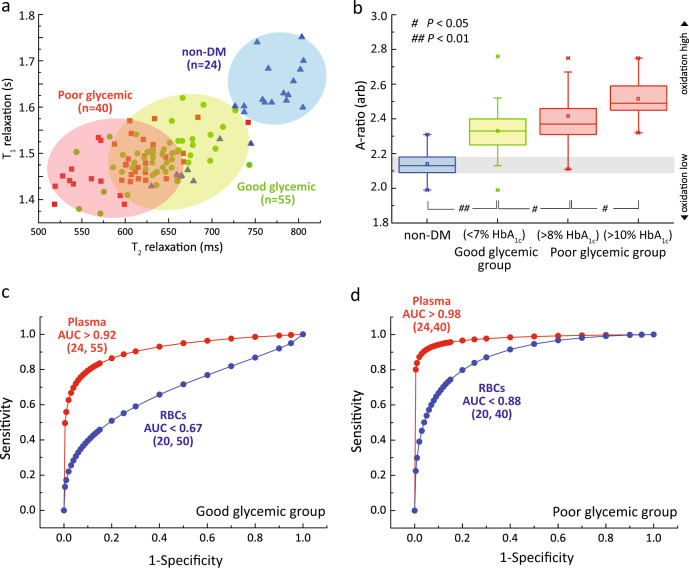
Baseline measurements of oxidative stress in plasma. **a** The *T*_1_–*T*_2_ relaxometry coordinates of plasma baseline taken from healthy non-DM subjects (blue, *n* = 24), subjects with good glycaemic control (green, *n* = 55) and subjects with poor glycaemic control (red, *n* = 40). **b** The corresponding A-ratio against the subjects with poor glycaemic control and good glycaemic control subgroups, as compared to healthy non-DM subjects. The subjects with poor glycaemic controls were further subdivided into >8% HbA_1c_ (*n* = 15) and >10% HbA_1c_ (*n* = 25) subgroups. The statistical significance was calculated using the Student’s T-test (two-tailed, unequal variance). The data is represented by box-plot format. The diagnostic accuracy of RBCs (blue) and plasma (red) taken from non-DM subjects and DM subjects, where their glycaemic subgroups were **c** good glycaemic control and **d** poor glycaemic control subgroups. The number of subjects (*n*) is indicated in parentheses.

Increased blood glucose promotes non-enzymatic glycation of plasma proteins, which include the albumin, alpha-crystalline, collagen, and low-density lipoprotein. Approximately half of the total serum protein is attributable to serum albumin^58,59^. Glycation and oxidative damage cause protein modification, which affects its functionality^60^. The micro MR analyses were performed at room temperature (26 °C). Each *T*_1_–*T*_2_ coordinate represents the composite redox properties of one subject’s plasma (Fig. 5a). The baseline readings of the DM subjects had, in general, much shorter *T*_1_ and *T*_2_ relaxation times, and it was well separated from the healthy non-DM subjects (blue). Notably, DM subjects with poor glycemic control, in particularly DM subjects with more than 10% HbA_1c_ subgroup (mean A-ratio of 2.52), saw a strong departure from the healthy controls (mean A-ratio of 2.13) (Fig. 5b).

The marked reduction in relaxation states was attributed to an increase in glycation and glycoxidation of the serum albumin, known as glucose toxicity. As a result of increased glycation (in vitro validation in Supplementary Fig. 8a) and protein oxidative damage^60^ (e.g., protein cross-linking), the mobility of bulk water proton was further restricted^61^, leading to reduction in *T*_1_ and *T*_2_ relaxation times. *T*_2_ relaxation however reduced much faster than *T*_1_ relaxation (and hence an increase in A_baseline_-ratio) (Fig. 5b). Similar trends were observed in vitro, which confirm the effect of glycation (Supplementary Fig. 8a, b) and glycoxidation (Supplementary Fig. 8c). A separate study by Cistola et al. recently found that the baseline *T*_2_ of water plasma showed a strong correlation in subjects with metabolic abnormalities^50,62^. Interestingly, the ROC analysis indicates plasma DM subjects with good glycemic control (AUC > 0.92) had much higher diagnostic accuracy than its’ counterpart RBCs (AUC < 0.67) (Fig. 5c), which suggest that pathological footprint of hyperglycemia is more prominent in extracellular plasma as compared to the intact RBCs. The same hypothesis is further observed and validated in DM subjects with poor glycemic control (Fig. 5d).

### Peroxide induced oxidation in plasma of DM subjects

**Fig. 6:**
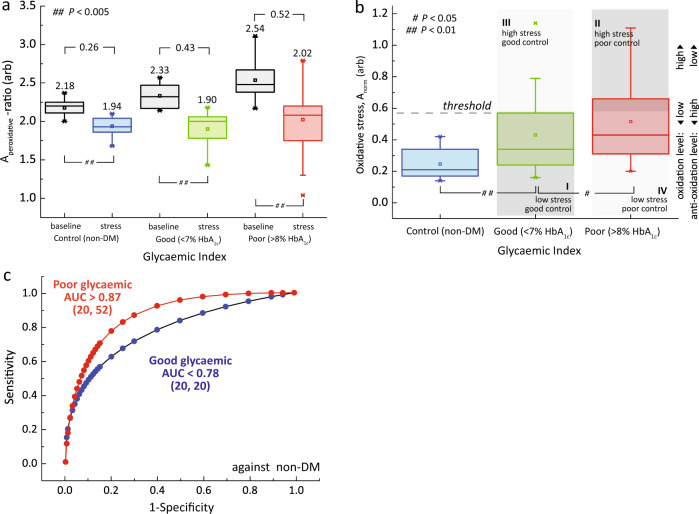
Peroxidative stress test on plasma. **a** The A_peroxidative_-ratio plot for plasma in baseline (black) and plasma pretreated with hydrogen peroxide (red, green, blue) for non-DM subjects (*n* = 20), and DM subjects with their glycemic index in good glycemic control (*n* = 20), and poor glycemic control (*n* = 52) subgroups. The means and changes in each subgroups were indicated above the box-plots. **b** A quadrant chart of diabetic subjects stratified into subgroups based on their peroxidative status (normalized A_baseline_–A_stress_) in association with their glycemic levels (e.g., HbA_1c_). The threshold of A_peroxidative_-ratio was set at 75th percentile that of DM subjects with good glycemic control, and adjusting based on clinical needs (e.g., population genetics). Note that the Y-axis (peroxidative stress) is in analogous with Y-axis (nitrosative stress) in quadrant shown in Fig. 4b. The details of quadrant representations are shown in Fig. 1e. The statistical significance was calculated using the Student’s T-test (two-tailed, unequal variance). **c** The diagnostic accuracies were evaluated using Receiver Operating Characteristic (ROC) for non-DM subjects against DM subjects with good glycemic control (blue) and poor glycemic control (red) subgroups. The number of subjects (*n*) was indicated on the parentheses.

In order to evaluate the total antioxidant capacity of plasma toward oxidation, we artificially challenged the plasma with hydrogen peroxide in subjects with poor glycemic control (*n* = 52), good glycemic control (*n* = 20), and healthy control (*n* = 20) (Fig. 6). Hydrogen peroxide solution was added into the freshly drawn plasma (10% v/v) for an incubation time of 10 min (Methods Online, Supplementary Fig. 9). The micro MR analyses were performed before (black) and after peroxide pretreatment for subjects with poor glycemic control, good glycemic control, and non-DM (red, green, and blue, respectively).

The peroxidative stress test revealed that a large spread phenotypic variations in DM subjects as compared to non-DM controls (Fig. 6a). The A-ratio for baseline measurements without stress (black) were in increasing trend (due to the effect of glycation, in Fig. 5)^63^. The effect of peroxidative stress (colored) resulted in a shift of plasma baseline (lower A-ratio). The normalized means (A_baseline_-ratio − A_stress_-ratio) were 0.26, 0.43, and 0.46 for non-DM, good glycemic, and poor glycemic control subgroup, respectively. This is due to the effect of glycoxidation which is markedly increased in DM subjects as compared to non-DM controls (Fig. 6b), in good agreement with in vitro validation (Supplementary Fig. 8b).

The proposed peroxidative susceptibility measurement (independent of HbA_1c_) can be used to further sub-stratify the DM subjects into smaller subgroups (Fig. 6b), in a similar fashion to RBCs (Fig. 4b). Exposure to peroxyl compound leads to an increased formation of disulfide bonds in albumin and human non-mercaptalbumin, which was also observed in several other pathological states^64,65^.

### Performance analysis of proposed stress marker

We demonstrate the clinical utilities and the sensitivity of the proposed technique by conducting observational, cross-sectional study. This study is designed to understand and evaluate the performance of the proposed biomarkers (oxidative status in RBCs) against the gold standard (oxidative stress in urine F_2_-Isoprostances (urinary F_2_-IsoP)).

**Fig. 7:**
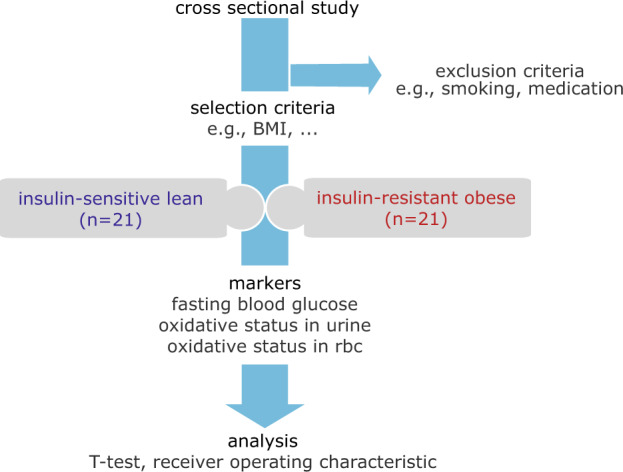
Study design: performance analysis of the proposed markers. **a** A cross-sectional, observational study was conducted to evaluate the proposed oxidative stress biomarker against the gold standard (urinary F_2_-IsoP). Subjects were recruited based on a number of selection (e.g., insulin sensitivity) and exclusion (e.g., smoker) criterion (details in “Methods”). Two distinctive group of subjects established were the insulin-resistant obese subjects (*n* = 21), and insulin-sensitive lean subjects (*n* = 21) with demographic as shown in Table 1. The biomarkers evaluated were oxidative stress in red blood cells (the proposed technique), oxidative stress in urine F_2_-IsoP, and fasting blood glucose.

Subjects’ recruitment was screened randomly and two subgroups were formed (demographic in Table 1) based on a range of selection criteria (e.g., insulin sensitivity, BMI) and exclusion criteria (e.g., smoking, on medication). This subgroup consists of subjects who are insulin-resistant obese and insulin-sensitive lean (Fig. 7). The subjects were recruited with prior written informed consent approved by the Institutional Review Board of National University Hospital. Fasting blood and urine sample were collected from the subjects. Functional stress analysis was performed on the RBCs using point-of-care technology (PoCT) NMR, and the urinary F_2_-IsoP analysis was performed using LC/MS (details in Method Online).

**Table 1:** Demographic of subject for recruited for the study. The statistical significance was calculated using the Student’s T-test (two-tailed, unequal variance).

	Lean (*n* = 21)		Obese (*n* = 21)		*T*-test
	Mean	(sem)	Mean	(sem)	*P*-value
Age (years)	23.3	0.16	28.6	0.64	<0.0005
BMI (kg m^−2^)	21.9	0.12	30.6	0.49	<0.0005
Weight (kg)	66.6	1.24	87.1	2.12	<0.0005
Height (cm)	174.1	1.77	168.7	1.87	0.04000
Waist circumference (cm)	79.7	0.37	101.0	0.65	<0.0005
Systolic blood pressure (mmHg)	110.0	2.40	120.0	1.44	0.001
Diastolic blood pressure (mmHg)	58.4	0.15	72.9	1.99	<0.0005
Fasting serum insulin (mmol/L)	4.8	0.26	19.3	0.85	<0.0005
HOMA-IR	0.9	0.05	4.1	0.21	<0.0005

**Fig. 8:**
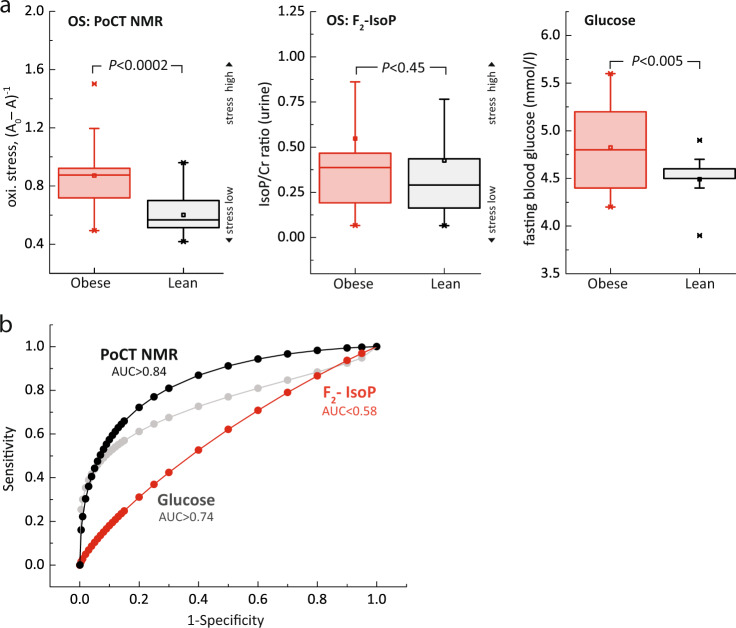
Performance analysis of the proposed markers. **a** The normalized concentration of oxidative stress (OS) in red blood cells, oxidative stress in urine F_2_-IsoP, and fasting blood glucose for obese subgroup (red) and lean subgroup (black). The oxidative stress in urine were corrected for urine creatinine concentration (F_2_-IsoP/Cr ratio). The statistical significance was calculated using the Student’s T-test (two-tailed, unequal variance). The data is represented by box-plot format. **b** The receiver operating characteristic (ROC) of the respective biomarkers; PoCT NMR (black), fasting glucose (gray), and urine F_2_-IsoP (red). Area under the curve (AUC) of larger than 0.8, 0.6, and 0.4 was considered as good, medium, and poor.

It appears that the oxidative stress in RBCs and urine were much higher in obese subgroup than lean subgroup (Fig. 8a). The level oxidative stress in RBCs (*P* < 0.0002) was, however, statistically far more significant than in urinary F_2_-IsoP (*P* < 0.45). The fasting blood glucose, expectedly, was significantly (*P* < 0.005) higher in obese subgroup than lean subgroup. Interestingly, the ROC for oxidative stress in RBCs (AUC > 0.84) was much higher than fasting blood glucose (AUC > 0.74) (Fig. 8b). A poor diagnostic accuracy (AUC < 0.58) was recorded for urinary F_2_-IsoP (Supplementary Table 2). This implies that the proposed marker has much higher predictive factor as compared to existing markers (e.g., fasting blood glucose), which is considered as one of the gold standard in the diagnosis of DM. However, extensive biomarker validation with population studies (large N) will further confirm and reveal many aspects of the biomarker, beyond the coverage of this paper.

## Discussion

We have developed a highly sensitive approach to accurately detect and quantify the redox (and hence oxidative/nitrosative) state and the subtle molecular motion changes of blood samples inferred based on the relaxation measurements. This is the first demonstration of the unique magnetic resonance relaxation properties of the various hemoglobin states, which were mapped out using the proposed magnetic state diagram. The measurement of redox properties in plasma/erythrocytes can provide a useful parameter for functional phenotyping of many biological pathways to better understand disease pathophysiology. This technology has vast potential to be applied for clinical disease diagnosis, prognosis, and monitoring, given that the specificity of the oxidative stress in association with the disease state can be further improved in the near future.

The platform presented here has several innovative features and is readily adaptable for clinical use (Supplementary Fig. 10). First, the miniaturized platform^31,66^ developed here is portable, and the proposed assays require minimal processing steps, low-cost, robust and can therefore be performed by a minimally trained operator. The high sensitivity can be attributed to the micron-sized detection coil and optimized ultra-short echo time implemented in this work. Only a minute amount of blood sample volume (<10 µL) is needed for each test, which enables the collection of sample using minimally invasive technique^31,67^ such as finger prick a standard procedure in patient care.

**Fig. 9:**
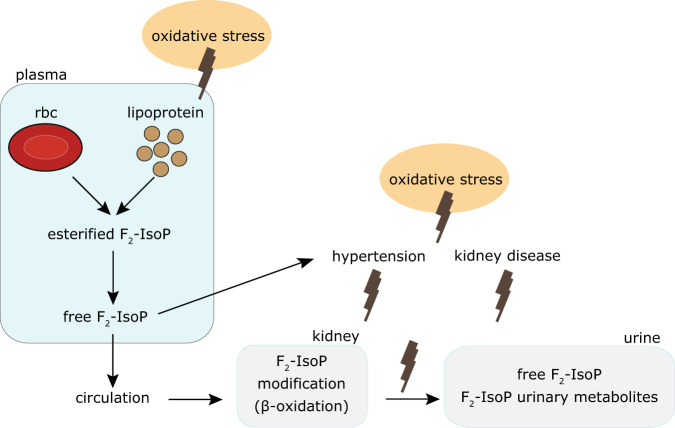
Schematic representation of the conceptualized mechanism of oxidative stress. Free F_2_-IsoP was formed from the esterified to phospholipids in RBCs and lipoprotein membranes in plasma. Free F_2_-isoP was then rapidly metabolized by the kidney and excreted in urine with its metabolites. Oxidative stress may affect the levels F_2_-IsoP directly but the downstream urinary F_2_-IsoP level may be modified indirectly by a series of events, including the kidney function, and hypertension.

Second, we exploited the non-destructive nature of magnetic resonance and introduced a number of in vitro functional assays that yielded parameters about the oxidative status of an individual, which may be clinically useful. It probes the primary redox event as compared to the current gold-standard biomarker, urinary F_2_-IsoP, which is a downstream marker and may be susceptible to confounding factors (Table 2). Oxidative stress may affect the levels F_2_-IsoP directly but the downstream urinary F_2_-IsoP level may be modified indirectly by a series of events (or confounding factors) including the kidney function and hypertension (Fig. 9).

**Table 2:** Comparison of salient features between the proposed method (PoCT NMR) benchmarking against the gold standard, urinary F_2_-IsoP.

	Proposed method	Gold standard
Biological liquid	RBC	Urine (isoprostance molecules)
Mechanism	Glutathione depletion	Lipid peroxidation
Method	Direct	Indirect
Functional test	Yes	No
Instrumentation	POCT NMR	LC/MS
Portability	Yes	No
Analysis time	Rapid (min)	Hours

The use of isoprostance as biomarker of oxidative status for correlation with disease outcome has so far yielded conflicting results in cross-sectional versus longitudinal studies^68,69^. Furthermore, they are static biomarkers that provide snapshots of the oxidative status of biological samples representing the in vivo condition of the subject at the point of collection. To accurately measure these molecules, laborious technique such as gas- or liquid-chromatography–mass spectrometry has to be employed, limiting its’ utility as diagnostic tools.

In summary, a new methodology for rapid manipulation and evaluation of the redox states in biological fluids (e.g., RBCs, plasma) using PoCT NMR is proposed. We demonstrated the clinical utilities of this technique to rapidly sub-stratify diabetes subjects based on their oxidative status in conjunction with their standard glycemic level. Through the use of the proposed methodology, we learnt new biological insights to the disease mechanism of DM, and discussed how sub-stratification of oxidative status may facilitate the early detection (or warning) of diabetes complications (e.g., cardiovascular event). We hope that the rapid and high-throughput analysis will fill up the gap between translational science and clinical medicine, and thus improving the overall outcome of clinical diabetes care and management.

## Methods

### Magnetic resonance relaxation measurement and detection

The proton nuclear magnetic resonance (NMR) measurements of predominantly the bulk water of red blood cells (RBCs) and plasma were carried out at the resonance frequency of 21.57 MHz using a bench-top type console (Kea Magritek, New Zealand) and portable permanent magnet (Metrolab Instruments, Switzerland), *B*_o_ = 0.5 T. A homebuilt temperature controller was constructed to regulate the temperature at 26 °C inside the measurement chamber. This helped maintain the stability of the magnetic field and biological sample under measurement. Single resonance proton micro magnetic resonance (MR) probe with a detection coil of 1.55 mm inner diameter was constructed to accommodate a heparinized microcapillary tube (outer diameter: 1.50 mm, inner diameter: 0.95 mm) (Fisherbrand, Fisher Scientific, PA) for a detection region of ~3.8 µL in volume. The longitudinal relaxation times, *T*_1_ were measured by standard inversion-recovery pulse sequences observed by CPMG train pulses. The transverse relaxation times, *T*_2_ were measured by standard CPMG train pulses (inter echo time: 200 µs) consisting of 2000 echoes. A total of 12 scans were typically acquired for signal averaging unless mentioned otherwise. The transmitter power output was maintained at 360 mW for a single 90^o^ pulse of 6 µs pulse length, which corresponds to a nutation frequency of 41.6 kHz. The delay between each pulse (recycle delay) was set at 1 and 4 s for RBCs and plasma, respectively.

### Ethics and blood collection

This study received approval from the local Institutional Review Board of the National Healthcare Group, and all participants provided informed consent. The EDTA-anticoagulated whole blood samples were collected using standard phlebotomy procedures. Fresh samples were used unless mentioned otherwise. For other non-critical analysis all blood samples were kept at ≤4 °C within two hours of collection and were kept refrigerated until analysis.

### Healthy subjects

Subjects without a past history of diabetes mellitus (DM) and had normal oral glucose tolerance test according to the American Diabetes Association criteria (fasting glucose <5.6 mmol/L; 2 h post oral glucose tolerance test glucose of <7.8 mmol/L) were recruited into this study following provision of informed consent. They were Chinese males aged between 21 and 40 years, with a body mass index below 23.5 kg/m^2^.

### Subjects with diabetes mellitus

Anonymized residual samples collected from DM patients at the outpatient clinic for measurement of glycated hemoglobin (HbA_1c_) as part of their clinical care, were included in this study. The HbA_1c_ was measured using the Bio-Rad Variant II analyzer. This National Glycohemoglobin Standardization Program (NGSP) certified instrument has an analytical coefficient of variation of <2% at HbA_1c_ concentration of 4 and 16%. Our laboratory is NGSP level 1-certified.

### Sample preparation and micro MR analysis

Fresh RBCs were washed three times with 1x PBS solution and resuspended at 10% hematocrit with PBS. The selected chemical was then mixed into the prepared blood at the desired concentration (see Biochemical Assays details below). The final concentrations were recalculated based on the entire volume. Other lower concentrations were prepared according to appropriate dilution. A horizontal shaker was used to homogenously mix (at 200 rpm) for all the chemically treated samples at room temperature. The blood was incubated between a few minutes to a few hours, as indicated in text. The blood was then washed three times to remove the chemical residual. A heparinized microcapillary tube is used to transfer 40 µL volume of blood via capillarity action. In order to obtain packed RBCs for micro MR analysis, the microcapillary tubes were spun down at 3000 *×* *g* for 1 min.

### Details on biochemical assays

#### Sodium nitrite treated RBCs

Twenty microliters of the desired concentration (in the range 500 µM to 100 mM) of sodium nitrite were then mixed into 180 µL of the prepared blood. *Hydrogen peroxide treated RBCs*. Twenty microliters of 3% hydrogen peroxide stock solution (~0.9 M), which was purchased commercially from Sigma-Aldrich, was mixed into 180 µL of the prepared blood. *Sodium salicylate treated RBCs*. Twenty microliters of the desired concentration (as described in the text) of sodium salicylic were then mixed into 180 µL of prepared blood. *Preparation of Oxoferryl Hb*. Oxoferryl Hb was prepared in two steps. The RBCs were first treated with sodium nitrite (similar to the protocol described above) to convert the RBCs into met-Hb. Hydrogen peroxide was then added into the met-Hb using the same protocol as described above. *Preparation of nitrosyl-Hb*. The nitrosyl-Hb was prepared in two steps. The RBCs were first converted into deoxygenated Hb (similar to the protocol described below) and treated with sodium nitrite using the same protocol as described above. *Preparation of deoxygenated Hb*. Twenty microliters of natrium hydrosulfite, Na_2_S_2_O_4_ (10 mM final concentration, Sigma-Aldrich) were then mixed into 180 µL of prepared blood and mix homogenously (at 200 rpm) for 10 min with a horizontal shaker. The UV-Visible spectrum was recorded immediately to confirm the presence of deoxygenated hemoglobin. Pure gas N_2_ was continuously purged into an airtight chamber in order to maintain the deoxygenated condition. The UV-VIS absorbance was used to confirm the presence of deoxygenated Hb by its distinct peak at 543 nm. *Hydrogen peroxide treated plasma*. The fresh whole blood collected was centrifuged at 14,000 *×* *g* for 5 min to separate the plasma from the packed RBCs. Ten microliters of hydrogen peroxide solution was then mixed into 90 µL of prepared plasma, and other lower concentrations were prepared according to appropriate dilutions.

### Performance analysis of proposed oxidative stress

#### Study design

Open-labeled, randomized, observational were carried out on insulin-resistant obese (*n* = 21) subjects and insulin-sensitive lean (*n* = 21) subjects. Fasting blood and urine sample were collected from the subjects. Oxidative stress analysis was performed on the RBCs using PoCT NMR, and the urine was frozen down before sending out for urinary F_2_-IsoP analysis with LC/MS.

#### Subject recruitment

We recruited 42 normoglycemic Chinese men (21–40 years; lean insulin-sensitive, *n* = 21 and obese insulin-resistant, *n* = 21). Exclusion criteria were history of smoking, thyroid disorder, malignancy, recent hospitalization, or surgery, first-degree relative with T2D, dyslipidaemia and its treatment, corticosteroids usage over the past 3 months, alcohol consumption (>3 units a day), moderate-to-high intensity physical activity (>5 h a week), or change in weight over the past 3 months (≥5%). The modified-WHO definition for obesity in Asians was used to define lean (18.5 ≤ BMI ≤ 23 kg/m^2^) and obese (BMI ≥ 27.5 kg/m^2^) subjects in this study. A Homeostatic Model Assessment-Insulin Resistance (HOMA-IR) score of <1.2 was employed for identification of insulin-sensitive lean subjects, and ≥2.5 for insulin-resistant obese subjects (Table 1).

### Ethics approval

Singapore’s National Healthcare Group Domain Specific Review Board (DSRB Ref. No: C/2013/00902) approved the study protocol, and Singapore Good Clinical Practice guideline and the principles of the 2013 Declaration of Helsinki were duly followed in performing all study procedures. Written consent was obtained from each subject before participation in this study.

### Oxidative stress F2-isoprostances measurements

Urinary free F_2_-Isoprostances were processed by anionic solid-phase extraction. Creatinine levels were measured to standardize the dilution of urine Photometric Analyzer (Roche Diagnostic GmbH, Germany). Samples were then measured by gas chromatography–mass spectrometry set at negative chemical ionization mode (Agilent Technologies, CA), with Triple-Axis Detector, connected to a gas chromatograph (Agilent Technologies, CA). Quantitation was achieved by comparing the peak area of free F_2_-isoprostanes with that of the relevant deuterated internal standard.

### Statistical analysis

Unless otherwise noted, all statistical analyses were performed using OriginPro (OriginLab Corporation, USA). For statistical analysis, *t*-tests were used. All error bars represent were either in standard deviation (s.d.) or standard error measurements (s.e.m.) of means and the statistical results were stated as *P*-values. The statistical significance was calculated using the Student’s T-test (two-tailed, unequal variance).

### Reporting summary

Further information on research design is available in the Nature Research Reporting Summary linked to this article.

## Supplementary information

Supplemental Information

reporting sum

## Data Availability

The data that support the findings of this study are available from the corresponding author (weng.kung@inl.int) upon reasonable request.
