# Supplementary material for: Molecular phenotyping of oxidative stress in diabetes mellitus with point-of-care NMR system
Source: NPJ Aging Mech Dis. 2020 Oct 5;6:11. doi: 10.1038/s41514-020-00049-0 (PMC7536436; doi:10.1038/s41514-020-00049-0)
Supplement: Supplementary file 1 — Supplemental Information [file 41514_2020_49_MOESM1_ESM.pdf]

Molecular Phenotyping of Oxidative Stress in Diabetes Mellitus with Point-of-care NMR system

Weng Kung Peng[1,2,3*], Lan Chen[2], Bernhard O Boehm[3,4,5*], Jongyoon Han[2,6,7*], Tze Ping Loh[8]

[1]Precision Medicine – Engineering Group, International Iberian Nanotechnology Laboratory, Portugal; [2]BioSystems & Micromechanics IRG (BioSyM), Singapore-MIT Alliance for Research and Technology (SMART) Centre, Singapore; [3]Lee Kong Chian School of Medicine, Nanyang Technological University, Singapore; [4]Ulm University Medical Centre, Department of Internal Medicine 1, Ulm University, Ulm, Germany; [5]Imperial College London, United Kingdom; [6]Department of Electrical Engineering and Computer Science, Massachusetts Institute of Technology, 36-841, 77 Massachusetts Avenue, Cambridge, MA 02139; [7]Department of Biological Engineering, Massachusetts Institute of Technology, 36-841,77 Massachusetts Avenue, Cambridge, MA 02139; [8]Department of Laboratory Medicine, National University Hospital, 5 Lower Kent Ridge Road, Singapore, 119074.

[*]Correspondence and requests for materials should be addressed to W.K.P. (weng.kung@inl.int), B.O.B. (Bernhard.boehm@ntu.edu.sg), J. Han (jyhan@mit.edu)

Supplementary Discussions

Calibration of micro MR analysis using spectrophotometry approach. Freshly drawn blood were washed, resuspended into 1*x* PBS and exposed to 6mM nitrite. The amount of oxy-Hb to met-Hb conversion was measured independently using micro magnetic resonance (micro MR) and spectrophotometry techniques at one-minute intervals. The kinetic profile of met-Hb formation over time (Supplementary Figure 1a) as monitored by micro MR correlated closely to the photospectormery technique (R^2>0.97) (Supplementary Figure 1b). The time-dependant oscillatory decay profile was also observed here, similar to the one described previously (Figure 2 in *Main Text*). It is worth noting that the sensitivity of the spectrophotometry measurement deteriorated (as indicated by the error bars) spectrophotometry as the met-Hb concentration reduced (Supplementary Figure 1b), suggesting low optical absorption properties of met-Hb. A summary Table of technology comparison with respect to our work is provided (Supplementary Table 1). We further investigated and compared the limit of detection for both the optical- and magnetic based methods (Supplementary Figure 2).

Limit of detection: Comparison between micro MR and spectrophotometry technique: Met-Hb were spiked into oxy-Hb at various concentrations and were measured using spectrophotometry analysis (Supplementary Figure 2) and micro MR system (Supplementary Figure 3). Repeat measurements (n=5) were performed for each concentration. The limit-of-detection (P-value<0.05) for spectrophotometry technique was found to be around 1% met-Hb for the three different biological samples. The optical spectra for low met-Hb (<1%) concentration were poorly resolved (Supplementary Figures. 2d), which was attributed to the low optical absorption of the oxidized Hb at 630 nm wavelength.

For micro MR analysis, the dilution experiement was repeated three times on blood samples obtained from three different individuals (samples A, B, and C). Paramagnetic susceptibilty of met-Hb causes reduction in T_1, T_2 relaxations and the A-ratio. The results in Supplementary Figure 3 suggest that a limit of detection (P-value<0.05) of approximately 0.0005% can possibly be achieved under well-controlled condition (Supplementary Figures. 3a, 3b, 3c). The proposed micro MR analysis was at least three to four orders more sensitivity than the spectrophotometry system used in this work. Note that, the sensitivity of micro micro MR analysis can be enhanced by introducing met-Hb with natrium fluoride to form fluoromet-Hb, which have much higher relaxivity and is stable towards temperature fluctuation[1].

Confounding factors. In the present study, we endeavored working on blood obtained freshly from subject, in particularly for clinical study in order to avoid possible confounding factors. These cells were then resuspended in 1*x* PBS for standardization. It is worth noting that there are a few possible confounding factors (*e.g.,* sample storage condition), which may affect the conditon of the blood samples. In particularly, we noticed that in red blood cells which are old (e.g., more than 1 week), will be affected the process of osmosis, *i.e.,* the movement of intra- and extracellular water and hence shifting the relaxations baseline. To demonstrate this effect, we artificially created a hypotonic environment to promote water movement into the cells. This was achieved by using diluted 1*x* PBS solution with 10% volume of DI-water, and hence forming final concentration of 0.9*x* buffer solution. As the cell gains water[2], the relaxation times (T_1=684.0 ms, T_2=182.6 ms) increased from their original relaxation states (T_1=621 ms, T_2=140.7 ms).

Low- and high-spin ferric hemoglobin in DM subjects. Oxidative stress is constantly produced endogenously and exogenously. Reactive oxidative species (ROS) is produced during oxygen metabolism and auto-oxidation, in which met-Hb is spontaneously produced. Most of met-Hb would be restored to their reduced state under normal physiologic condition. However, as cells aged[3] or are under constant pathological stress[4], the distal histidine of the Hb will bind to the iron and denature to form hemichrome (HC). The irreversible hemichrome will eventually aggregate and precipitate on cellular membrane. However, the actual physiological roles of hemichrome remains controversial[5]. While Hb promotes most of the biological oxidative processes, oxidized Hb (*e.g.,* HC, met-Hb and free heme) can be a source of stress themselves, and cause damage to RBCs membrane and cytoplasm, and functional impairments.

HC can be induced *in-vitro* by incubating sodium salicylate with freshly collected blood and washed three times before micro MR analysis (Methods Online). HC was poorly resolved with a broad valley between 540-580 nm in spectrophotometer[6]. In contrast, the micro MR analyses indicated that high-spin met-Hb (T_2=98ms, T_1=198ms, A=2.02) and low-spin HC (T_2=113ms, T_1=587ms, A=5.19) had unique relaxation times (and hence, the A-ratio). Although HC has similar ferric oxidation state as met-Hb, it is in a much lower spin-state no unpaired electrons (Figure 1e). As the distal histidine of the Hb binds to the iron on the sixth coordinate, the T_1 for HC is therefore significantly longer than T_1 of met-Hb, which has water proton on the sixth coordinate, and therefore subject to different Brownian fluctuation. Thus, despite sharing the ferric-oxidation state, both the HC and met-Hb can be resolved based on their magnetic properties (as compared to optical properties) as reflected in the state-diagram (Figure 3b and Supplementary Figure 4)

Nitrite concentration selection criteria for nitrite—induced oxidation of RBC assays (short-time 10 min incubation). We investigated the optimal nitrite concentration (for an incubation of 10 min) to be used in our stress test. The optimal concentration (e.g., within the redox homeostasis viable range) must be able to reflect the highest degree of inter-individual variability to improve the resolution. Twelve subjects were randomly selected from both the good and bad glycaemic control groups. Freshly drawn blood samples were washed and resuspended in 1*x* PBS. The blood samples were then treated with various concentration of sodium nitrite for 10 min (Supplementary Figure 5a) and the micro MR analyses were performed (Supplementary Figure 5b).

Several criteria were used to determine the optimal nitrite concentration. Firstly, the stressed samples (blue) must be significantly different (P-value<0.05) from their respectively baselines (orange bar, in Supplementary Figures. 5b-c). Secondly, it is desirable to have high degree of inter-individual variability that reflect high resolution as manifested by the large spread of the box plots. Nitrite concentration in the range of 2 mM to 8 mM, reflected the bio-homeostatis and satisfied these prerequisites (Supplementary Figure 5d). Nonetheless, higher level of differentiation (resolution) between subjects can be achieved by induction with much higher nitrite concentration. In this work, 6 mM nitrite (unless otherwise specified) was chosen for the nitrite-induced oxidation assay.

Nitrite concentration selection criteria for nitrite—induced oxidation of RBC assays (long-time 36 hrs incubation). The same experiment as above was repeated on five randomly chosen subjects across a range of HbA_{1c} (5.4%—13%) and their responses to various nitrite concentrations (0, 5 µM, 0.01 mM, 0.1 mM, 2.0 mM, 4.0 mM) were recorded (Supplementary Figure 6). In this experiment, the incubation period was increased to 36 hrs from 10 min (Supplementary Figure 5). To drive a complete inversion from oxy-Hb to met-Hb, we found that only 4 mM nitrite required instead of 10 mM. For a longer incubation time (*i.e.,* 36 hrs), the homeostatis feedback range has shifted to 0.5 mM—2 mM instead of 2 mM to 6 mM, as shown previously. Inter-individual variability at low nitrite concentration (µM range) appeared to be much smaller as depicted in log-log plot in Supplementary Figure 6b.

Prolonged effect of *in vitro* exposure to peroxidative stress on RBCs. Perodixe is a byproduct of oxygen metablolism, which produces intracellular reactive oxygen species[7]. The steady state concentration of H_2O_2 in the RBCs is reported to be approximately 0.2 nM[8], and a dramatic increase by autoxidation is possible[9]. We investigated the effect anti-oxidant capacity of RBCs due to prolonged (day to weeks) *in vitro* exposure to low concentration (90 nM) of hydrogen peroxide (H_2O_2) (Supplementary Figure 7).

Five subjects with HbA_{1c} from 6.1% to 13.8% were randomly selected for the this experiment and the anti-oxidant capacity of the RBCs were measured by nitrite-induced oxidation assay. Freshly drawn blood samples were washed and resuspended in 1*x* PBS (Day 0) and divided into two groups; one pretreated with H_2O_2 and a control (without treatment). In order to understand the total intracellular anti-oxidant properties of RBCs, rather than the whole blood, the plasma was removed. The micro micro MR analyses were performed at Days 1, 6, 13 of post exposure using the nitrite-induced oxidation assay to induce intracellular anti-oxidant capacity of the RBCs. On Day 1 post exposure, both the control and peroxide-treated groups had similar measurements ($P > 0.05$) (Supplementary Figure 7a). However, on Day 6 (Supplementary Figure 7b), two of the five subjects in the peroxide-treated group (blue) registered a drop in their anti-oxidant capacity as compared to control (red).

The same experiment was repeated but with five folds increase in hydrogen peroxide concentration to 500 nM. On Day 1 of post-exposure, a drop in anti-oxidant capacity was registered in all five subjects (Supplementary Figure 7d) and was much more significant than previously observed (Supplementary Figure 7b—c). One can interpret this *in vitro* observation to understand the *in vivo* cellular damage. While acute infection and impaired ability to reproduce enzymes in congenital diseases are known to have averse effect, slightly elevated stress above normal physiological condition over a prolonged period (*e.g.,* chronic diseases, unhealthy diet) can result in reduction anti-oxidant capacity. Under normal physiological condition however, cells would be able to constantly repair and renew themselves provided that appropriate nutrition is available.

***In vitro* glycation of plasma and oxidative status:** In order to investigate the effect of glucose on plasma, we performed *in vitro* glycation by adding glucose (control, 10mM and 100mM) to freshly drawn plasma on three different subjects (Day 0). The micro MR analysis, spectrophotometry absorbance, and glucose level were recorded simultaneously on day six after incubation. The initial plasma glucose readings were 5.6, 7.1, 7.3 mmol/L for subject A, B and C, respectively.

On Day 6, the T_1 and T_2 relaxations showed marked reduction as glucose was artificially introduced in the plasma samples of the three subjects (Supplementary Figures. 8a—b). T_2 relaxation reduced much faster than T_1 relaxation (Supplementary Figure 8a), which increased the $A_{baseline}$-ratio (Supplementary Figure 8b-c) and was due to the effect of glycation. This corresponded well with the cross-sectional study (*in vivo* measurements shown in Figure 5a, and Figures. 6a—b in the main manuscript). Peroxidative assay with 0.3% hydrogen peroxide revealed that plasma has a much lower antioxidant capacity as a function of glycemic level (red box in Supplementary Figure 8b). As more plasma is oxidized, the T_1 relaxation time reduces much faster than T_2 relaxation time, and

hence the reduction in $A_{peroxidative}$-ratio (Supplementary Figure 8b). Despite the fact that both glycation and glycoxidation reduced T_1 and T_2 relaxation times, the $A_{peroxidative}$-ratio (red) and $A_{baseline}$-ratio (black) were orthogonal parameters (Supplementary Figure 8b) making it possible to differentiate between the two effects.

The changes of A-ratio due to peroxidative stress were significant large (P<0.05) (0.29, 0.34, 0.40) for (control, 10mM, 100mM), respectively. The *in vitro* study here allows one to quantitatively study the longitudinal effect of glucose exposure on the same subject by simulating the *in vivo* condition. Furthermore, we observed that the effect of *in vitro* glycation and glycoxidation were similar as revealed in the cross sectional *in vivo* study (Figures. 5 and 6). It is also worth noting that micro MR analysis of 5.5 mM of glucose with and without 0.3% H_2O_2 mixture (10% v/v) were T_2=2290ms, T_1=2441ms, A=1.07 and T_2=2337 ms, T_1=2461 ms, A=1.05, respectively. This implied that either H_2O_2 did not interact directly with glucose (no physical and color changes or precipitate were observed) or it did not alter the relaxation states substantially.

Peroxide concentration selection criteria for peroxide-induced oxidation of human plasma assays. We investigated the optimal peroxide concentration to be used in our stress test. The optimal concentration (e.g., within the redox homeostasis viable range) must be able to reflect the highest degree of inter-individual variability providing the highest resolution. The subjects (n=8) were randomly selected from both the good and poor glycaemic control groups. Firstly, the stress samples needed to be statistically significant (P < 0.5 and AUC > 0.8) from its' respecitve baseline. Secondly, it is desirable to have large degree of inter-individual variability, as indicated by the spread between 25% and 75% quantile box plots (Suplementary Figure 9). The 0.3% to 0.4% peroxide concentration range fit the selection guidelines. In this work, we have choosen 0.3% peroxide, unless mentioned otherwise in the manuscript.

Evaluation of analytical and sampling variations: The consistency of the measurement (e.g., reproducibility) is a function of the biological samples (the time-scales of the relaxation properties) and instrumentation (analytical) stability. To demonstrate the effect of analytical and biological variations, we conducted a simple validation (Supplementary Figure 10a). Five repeated measurements were performed on the same sample inserted, and repeated the same procedure on the second sampling (same sample of different vials). The T_1 and T_2 measurements were performed on both the plasma and RBCs.

As evaluated by the *Student T-Test* analysis, the statistical variations were insignificant (P >0.05) for all the cases (A, B, C, D) reported, which suggested that the reproducibility of the between the first and second samplings were very high. Secondly, we observed that the variances (uncertainty error) from the repeated measurements, which presumably reflecting the instrumentation stability and to certain extent the biological fluctuation process were (typically) less than 6%. Therefore, as described by the procedure proposed above, one single sampling is sufficient to represent the entire sample, provided if the sample is well-mixed (via rigorous pipetting). Nonetheless, multiple sampling is encouraged if there is no time-constraint.

Inter and intra-individual Variability. In the interest of clinical application, we evaluated the intra- and inter-individual variations, which reflects the reliability and the consistency the methodology proposed here. Five healthy individuals (Subject 1 to 5) were arranged for blood donation (after overnight fasting condition) thrice in every successive week. T_1 and T_2 measurements were performed thrice on each sampling blood every week (Supplementary Figure10b). The weekly repeated measurements were highly consistent and stable as shown in the results where the readings from the same subjects clustered together. The intra individual coefficient variations were less than 2.5% and 4.5% for T_1 and T_2 readings respectively. In addition, the inter-individual variability is much larger than intra variability which indicate that the present method can be used a stable marker. The results indicated that the micro MR analyses were highly time- and patient-specific, making it suitable for clinical application.

The raw decay signals: Human serum albumin (HSA) accounts for approximately 50% of total plasma proteins. HSA contains 35% of cysteine residues that form redox reactive disulfide bridges (S-H), which plays an important role in increasing the endogenous antioxidant capacity[10]. The disulfide bonds can be reduced and oxidized depending on the net exogenous (or endogenous) electron, which are supplied (using the H_2O_2 assay). In this study, the plasma were collected from subjects who had fasted (>8 hours) from the previous night, so that only the endogenous properties of the serum in the patients in the native state (e.g., without the confounding factor of food intake) is measured. From the raw data (Supplementary Figure 10c), it appears that the T_1 and T_2 relaxation signals for plasma exhibit a mono-exponential decay. The sample stability as the function of time in 4^0C storage were investigated and was found to be less than 5% variations (Supplementary Figure 10d).

We noticed however, that the T_2 relaxation for RBCs were not a complete fit with mono-exponential fitting (Supplementary Figure 10c (iv)). This may be due the presence of a macromolecular proton

(e.g., immunoglobulin, lipoprotein, and lipid) with fast relaxation component. Nonetheless, mono-exponential fitting gave a compromised weighted average of possibly multiple relaxation rate. Phenomenologically, we observed that the *in-vivo* calibration of RBCs (Figure 2 in *Main Text*) were consistent and mono-exponential fitting had served our purpose sufficiently.

We postulate that there may be other biological samples that are non mono-exponential in nature, and hence, the current simplification method will have to be treated with caution (case by case basis). Therefore the use of higher order analysis, such as the use of multidimensional Inverse Laplace decomposition[11], which can yield higher accuracy (and resolution) in the time-domain spectra can be used. It is expected that each decomposed relaxation rates can then form an individual T_1-T_2 coordination mapping (and their respective T_1/T_2 ratio) of their specific relaxation reservoirs and the current demonstration in this work represents the basis of the higher order analysis.

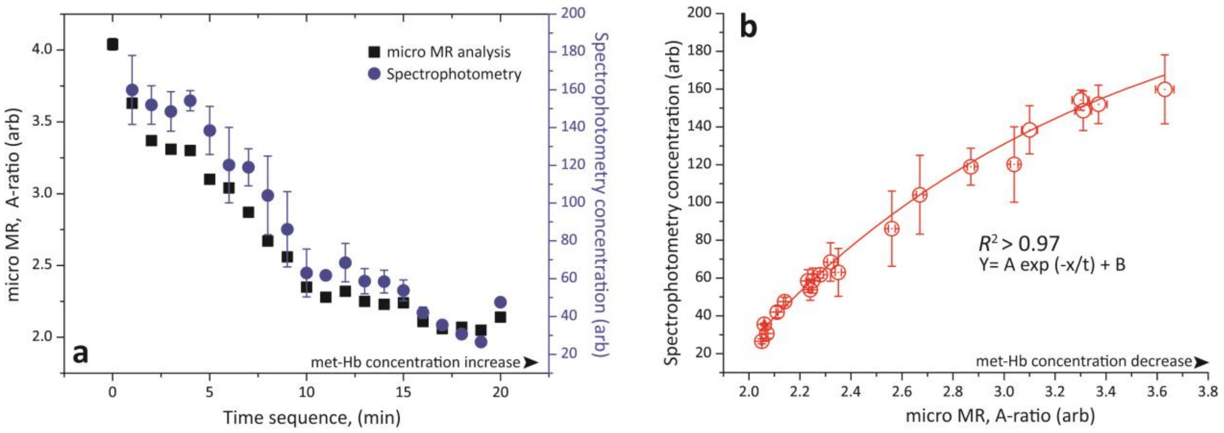

Supplementary Figure 1a: Calibration of micro MR using spectrophotometry approach. (a) Time dependent kinetic profile for nitrite—induced oxidation of oxy-Hb as measured by micro MR (n=3) and spectrophotometry (n=5 to n=8). 6 mM nitrite concentration was used and the RBCs were sampled and measured at every 1 min interval. Error bars were standard deviation. Note that the error bars for micro MR analyses were too small (<5%) and hence does not appear visible in the plot. (b) Plot between spectrophotometry readings against the micro MR analyses.

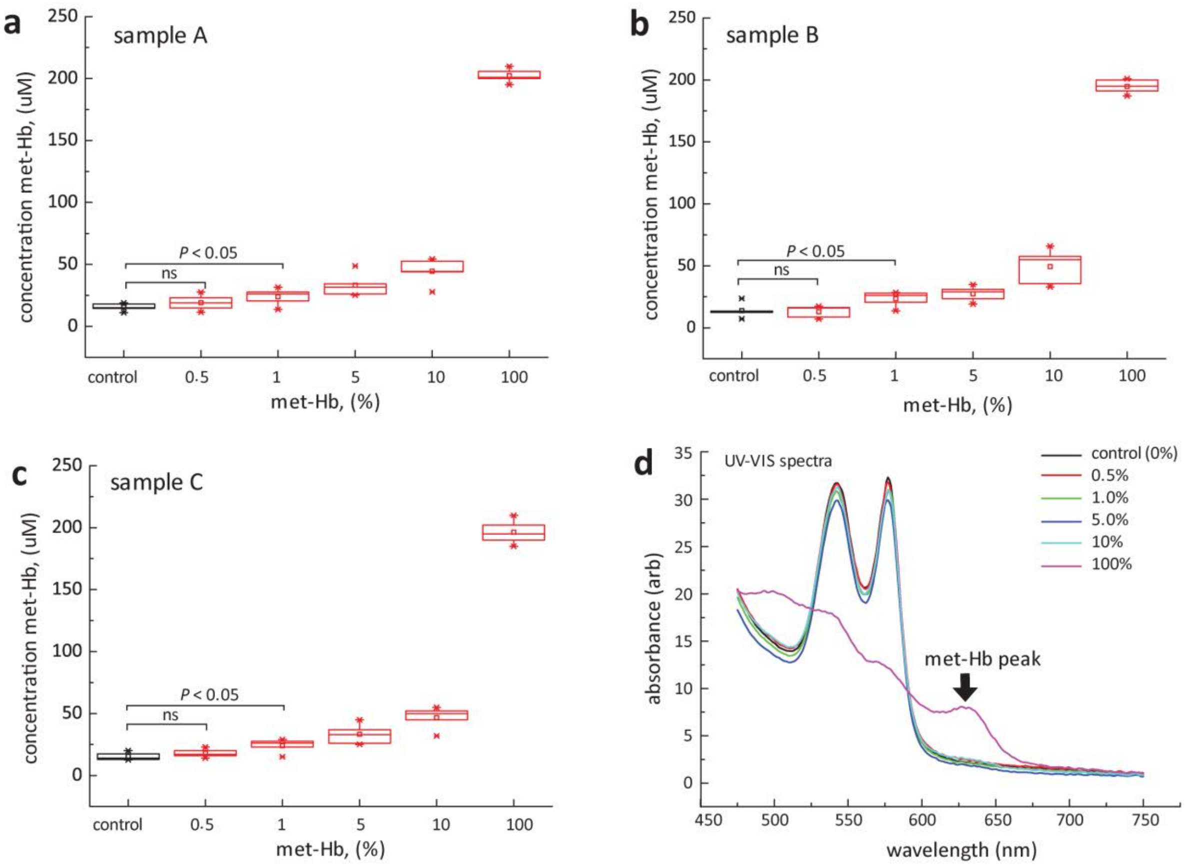

Supplementary Figure 2: Limit of detection for spectrophotometry technique in met-Hb detection. Spectrophotometry spectra for met-Hb dilution (with oxy-Hb) in various concentrations (e.g., 0.5%, 1%, 5%, 10%, 100%) taken from (a) donor A, (b) donor B, and (c) donor C, and the (d) UV spectrum. Multiple samplings (n=5) were carired out for every order of dilution. One tailed student's t-test of paired test were used to calculate the *P*-value. The concentration (μM) were calculated from two absorbance peaks at 577 nm and 630 nm and based 1 mm optical path[12], the [oxy-Hb]=$6.6A_{577}$—$8A_{630}$ and [met-Hb]=$27.9A_{630}$—$0.3A_{577}$.

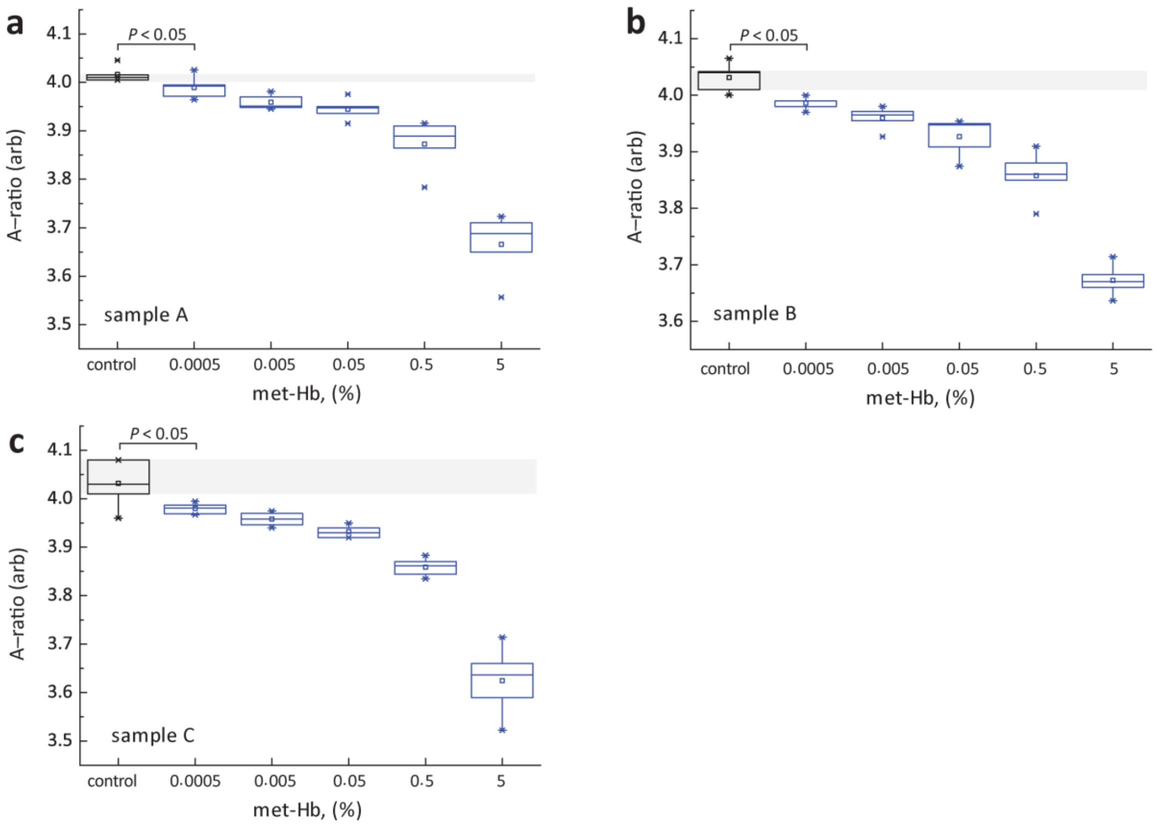

Supplementary Figure 3: Limit of detection for micro MR analysis in met-Hb detection. T_1—T_2 state diagram (left panel) and A—ratio against the met-Hb concentrations (e.g., control, 0.0005%, 0.005%, 0.05%, 0.5%, 5%) for (a) sample A, (b) sample B, and (c) sample C. Multiple samplings (n=5) were carried out for each order of dilution. One tailed student's t-test of paired test were used to calculate the *P*-value.

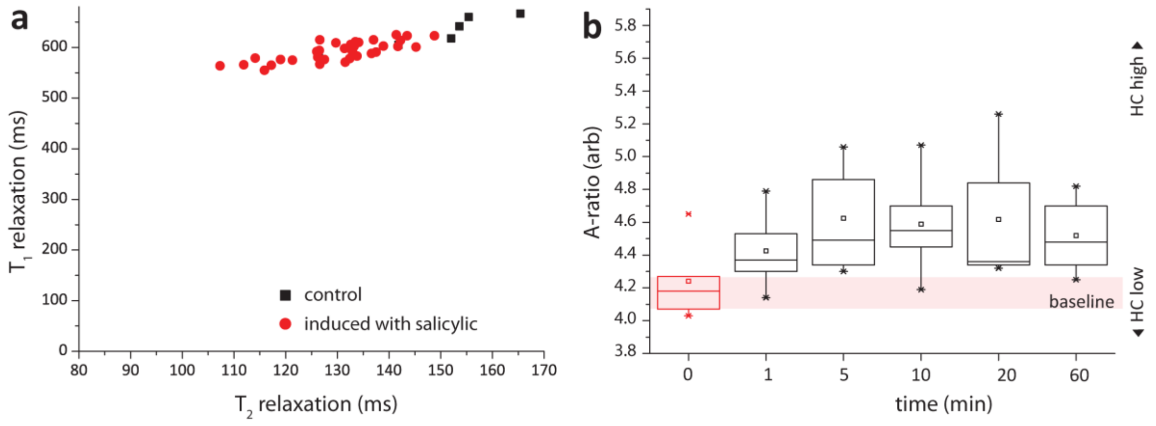

Supplementary Figure 4: Low-spin ferric hemoglobin (hemichrome) in DM subjects. The RBCs were chemically induced with 0.125 M sodium salicylate (red) at several intervals (1, 5, 10, 20, and 60) minutes and compared to that of controls (black). (a) The T_1-T_2 state diagram and its´ corresponding (b) A-ratio plot as a function of time.

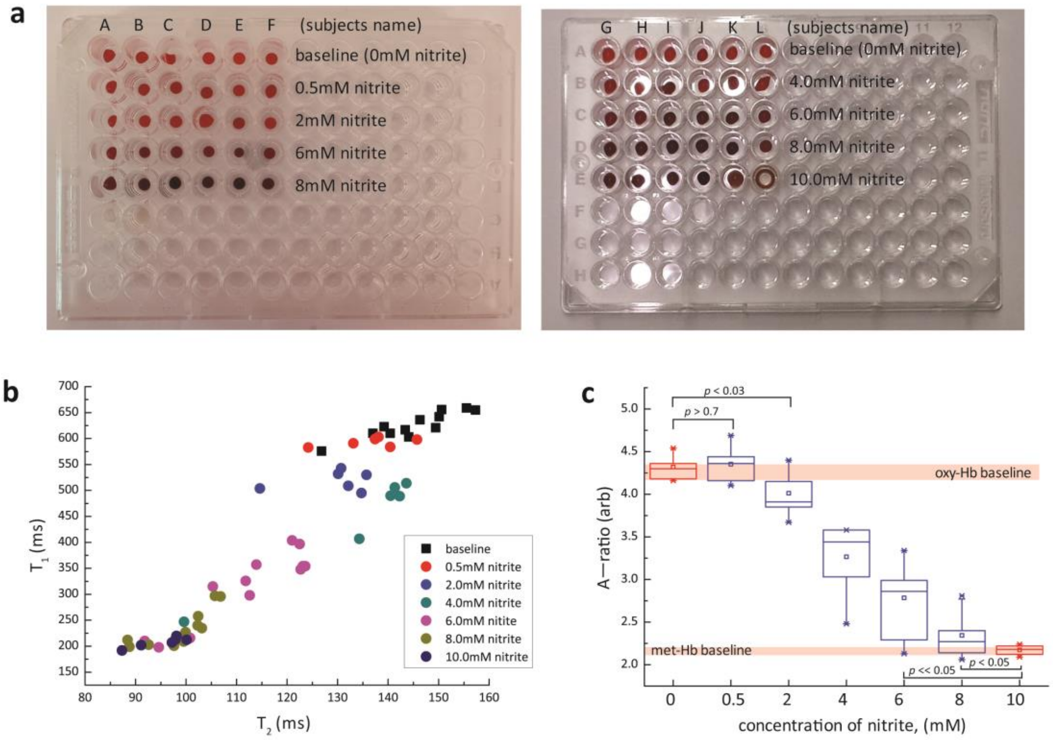

d

	ROC-AUC (nitrite concentration)						
	0mM	0.5mM	2.0mM	4.0mM	6.0mM	8.0mM	10mM
oxy-Hb baseline	-	0.444	0.875	1	1	1	1
met-Hb baseline	1	1	1	1	0.95	0.736	-

Supplementary Figure 5: Nitrite concentration selection criteria for nitrite—induced oxidation assay (short time 10 min incubation). (a) The actual micrograph of the subjects' blood with various nitrite concentrations. (b) Met-Hb formation indicated by the A—ratio of randomly assigned subjects (n=12) taken from a range of HbA_{1c} between 4.2% to 9.7% and their responses over various nitrite stress concentration (0, 0.5mM, 2.0mM, 4.0mM, 6.0mM, 8mM, 10mM) on RBCs. (c) The corresponding A-ratio plot as a function of nitrite concentration. Box plot indicate the 25% and 75% quantiles. The *P*-values were calculated by using student's t-test for two samples-test of unequal variances. (d) ROC—AUC calculated for each nitrite concentration against the oxy-Hb and met-Hb baseline. AUC of >0.9, >0.8, <0.7, is consired as excellent (dark blue), good (blue) and poor (light blue), respectively.

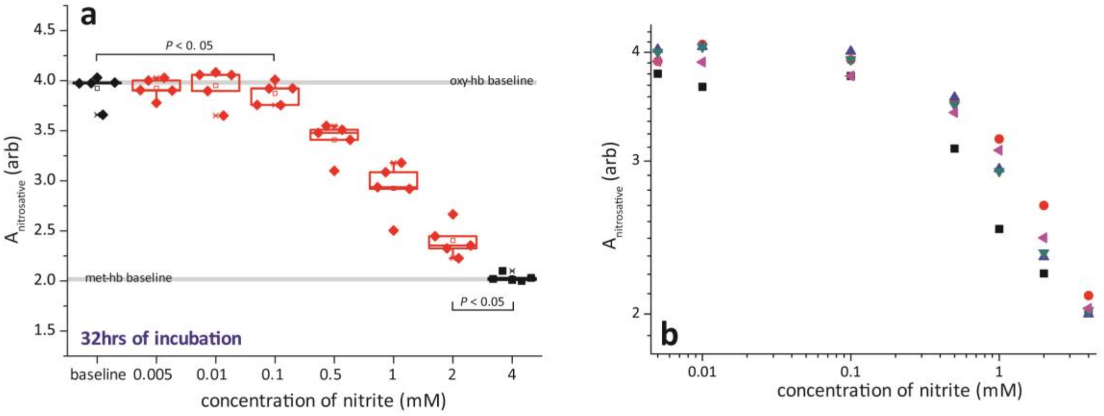

c

	ROC AUC: nitrite conentration (mM)							
	**0**	**0.005**	**0.01**	**0.1**	**0.5**	**1**	**2**	**4**
oxy-hb baseline	-	0.56	0.36	0.68	1	1	1	1
met-hb baseline	1	1	1	1	1	1	1	-

Supplementary Figure 6: Nitrite concentration selection criteria for nitrite—induced oxidation assay (long time 32 hrs incubation). (a) Met-Hb formation indicated by the A—ratio of randomly assigned subjects (n=5) for nitrite concentration (0, 5μM, 0.01mM, 0.1mM, 2.0mM, 4.0mM) on RBCs. The subjects were of HbA_{1c} of between 5.4% to 13%. (b) The corresponding A-ratio plot as a function of nitrite concentration. Box plot indicate the 25% and 75% quantiles. The *P*-values were calculated by using *student's t-test* for one-tailed test of unequal variances. (c) ROC—AUC calculated for each nitrite concentration against the oxy-Hb and met-Hb baseline. AUC of >0.9, >0.8, <0.7, is considered as excellent (dark blue), good (blue) and poor (light blue), respectively.

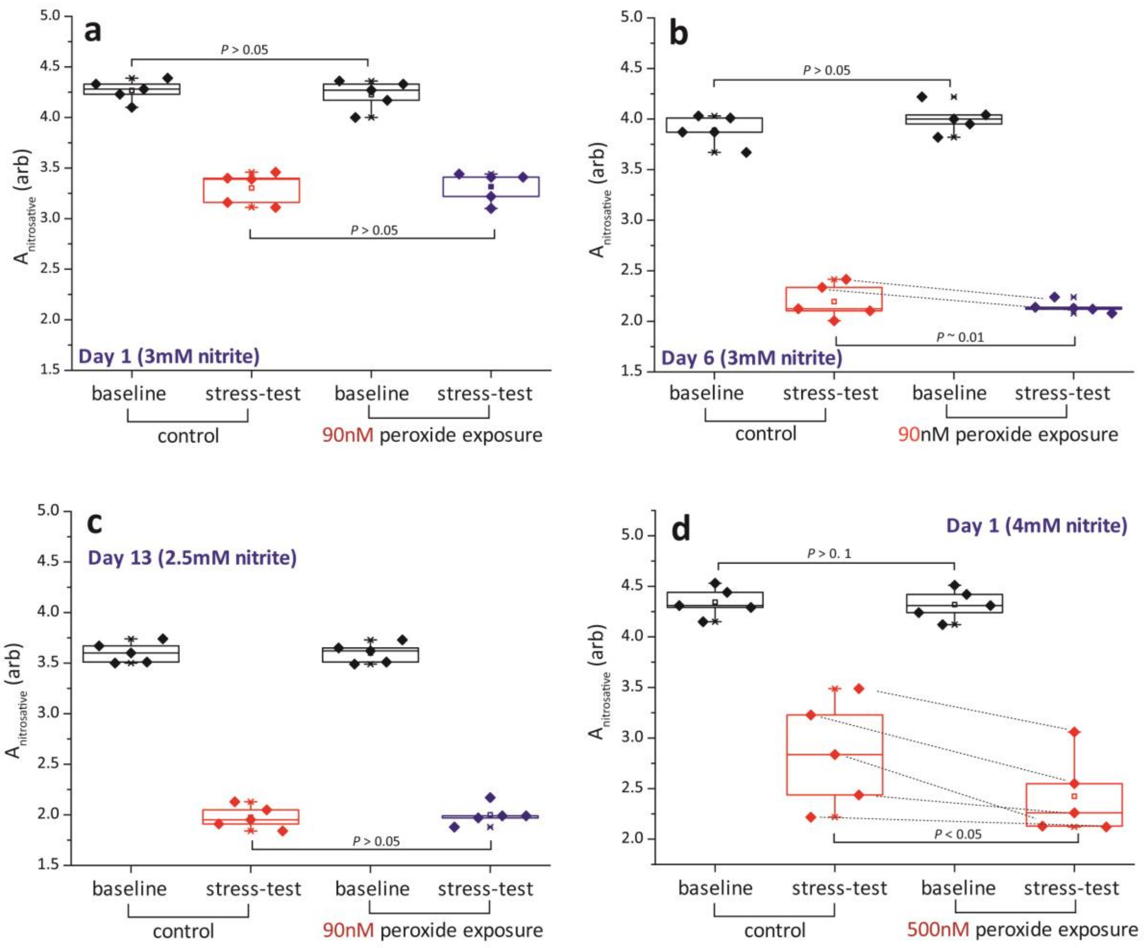

Supplementary Figure 7: Long term *in vitro* exposure to 'mild' concentration of H_2O_2. Randomly selected diabetic subjects (n=5) taken from a range of HbA_{1c} (4.8% to 11%). The blood from the same donor was separated into two groups: with and without exposure to 90 nM of hydrogen peroxide. The period of incubation is indicated in Day 1, Day 6 and Day 13. Their responses to nitrite—induced oxidation were then measured, for the baseline without nitrite-stress (black) and after oxidation (red, blue) RBCs. Concentration of nitrite used against the met-Hb concentration (in A—ratio) on (a) Day 1 (3 mM nitrite), (b) Day 6 (3 mM nitrite), (c) Day 13 (2.5 mM nitrite). (d) The same experiment was repeated but with increased 500nM of hydrogen peroxide. Measurement on Day 1 (4 mM nitrite). Box plot indicate the 25% and 75% quantiles. The *P*-values were calculated by using *student's t-test* for one-tailed paired test. The arrow indicate the matching reading taken from the same subject.

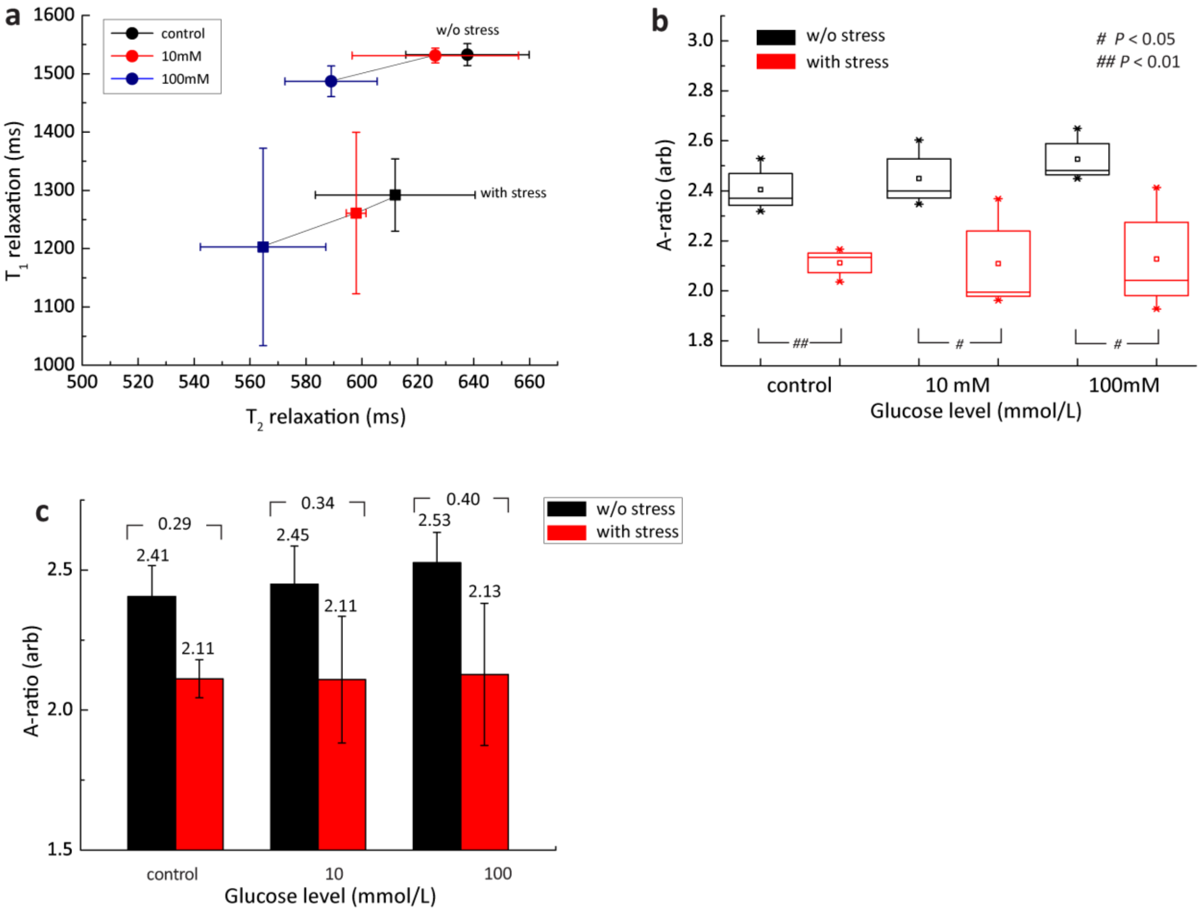

Supplementary Figure 8: *In vitro* glycation of HSA and oxidative status. (a) The T_1—T_2 state diagram of *in vitro* glycation with glucose (0 mM (black), 10 mM (red), and 100 mM (blue)) baseline w/o stress and with peroxidative stress. The error bars are standard error measurements (s.e.m) of three different subjects (n=3). The statistical differences were calculated using Student T-Test indicated in all the sub-groups. The breakdown glucose level before (black) and after (red) stress were shown in (c). The number indicated are the respective A-ratio (and its´ changes).

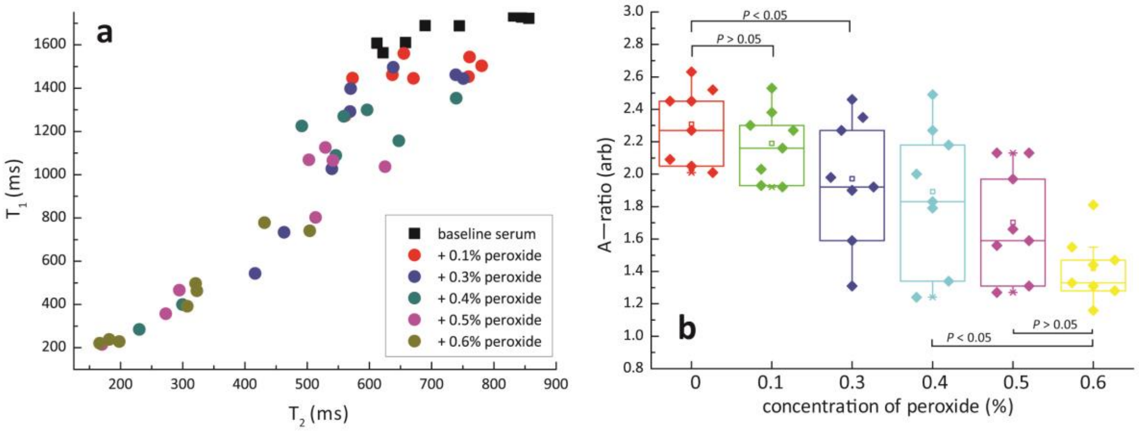

c

	ROC-AUC (Peroxide concentration)					
	0	0.10%	0.30%	0.40%	0.50%	0.60%
baseline	-	0.64	0.8	0.81	0.9	1
baseline (oxidized)	1	1	0.9	0.82	0.76	-

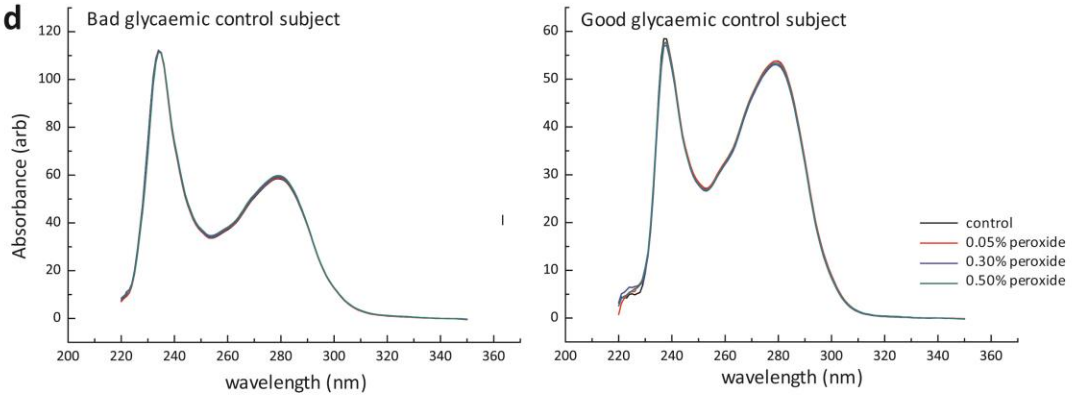

Supplementary Figure 9: Peroxide concentration selection criteria for peroxide—induced oxidation of human plasma assays. (a) T_1- T_2 plot of human plasma taken from randomly assigned subjects (n=8), and its' responses as a function of peroxide concentration (0, 0.1%, 0.3%, 0.4%, 0.5% and 0.6%). (b) The corresponding A-ratio plot, as a function of hydrogen peroxide concentration. Box plot indicate the 25% and 75% quantiles. The *P*-values were calculated by using student's t-test for one tailed of unequal variances. (c) ROC—AUC calculated for each peroxide concentration against the non-oxidized and non-oxidized plasma baseline. AUC of >0.8 and <0.7, is consired good (dark blue), and fair (blue), respectively. (d) Spectrophotometry spectra of plasma taken from 2 subjects at various peroxide concentrations (0.05%, 0.30% and 0.50%) against controls.

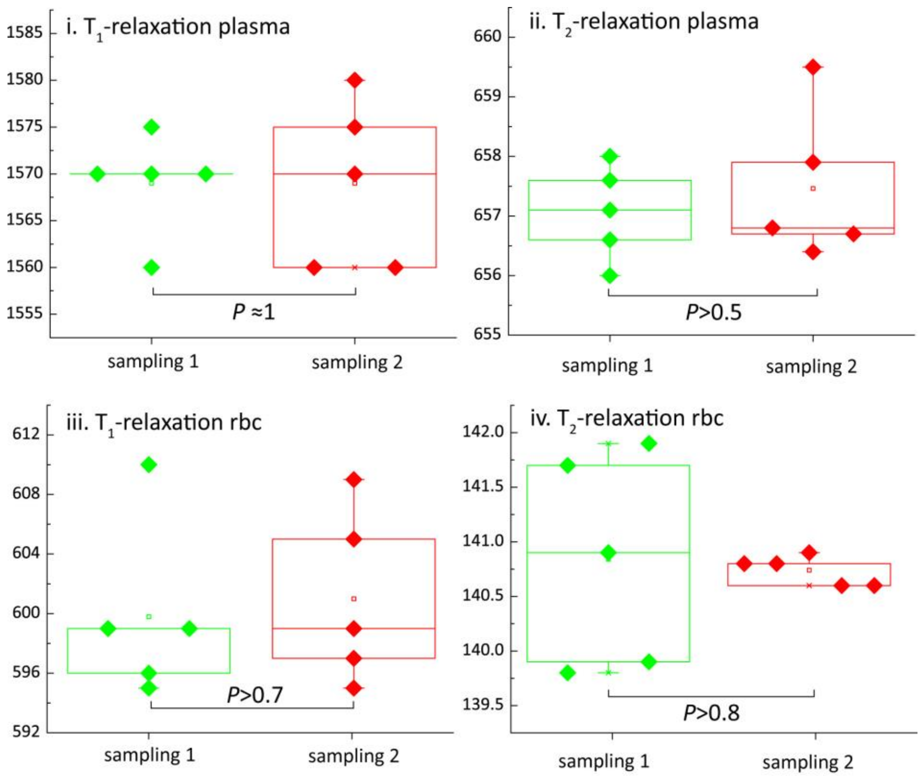

Supplementary Figure 10a: Evaluation of analytical and biological variations. (i) T_1-relaxation, (ii) T_2-relaxations for plasma, and (iii) T_1-relaxation, (iv) T_2-relaxation for red blood cells. Each point represent single measurement. The intra-assay coefficient variations (% of variance against the means) were (i) (1.9%, 0.095%), (ii) (5.1%, 0.25%), (iii) (5.6%, 0.01%), (iv) (5.95%, 0.68%), respectively. The pulse sequence used were as reported on *Methods Online*. The statistical significant were calculated using two-tailed T-Test of equal variance.

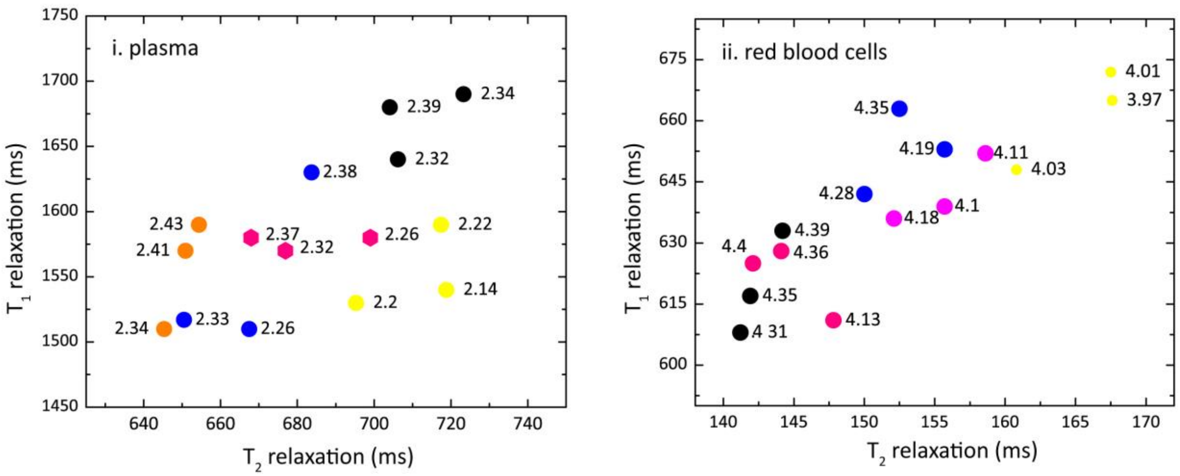

Supplementary Figure 10b: Inter and intra-individual variability. T_1-T_2 relaxation diagram for (a) plasma, and (b) RBCs. Each point represent the mean reading of three samplings taken from the same day. The A-ratio is indicated in the label. Each subject is represented by the same color, *e.g.,* in the case of plasma, the subjects were Subject 1 (orange), Subject 2 (blue), Subject 3 (black), Subject 4 (yellow) and Subject 5 (red). The number were randomly assigned and the measurements were carried out blinded. The intra individual coefficient variations (i) (1.5%, 1.6%), (2.5%, 4.3%), (1.9%, 2.1%), (0.7%, 2.7%), (2.3%, 0.4%), (ii) (1.1%, 2.1%), (1.9%, 1.6%), (2.1%, 1.3%), (2.4%, 1.8%), (2.0%, 1.5%) for Subject 1 to Subject 5, respectively.

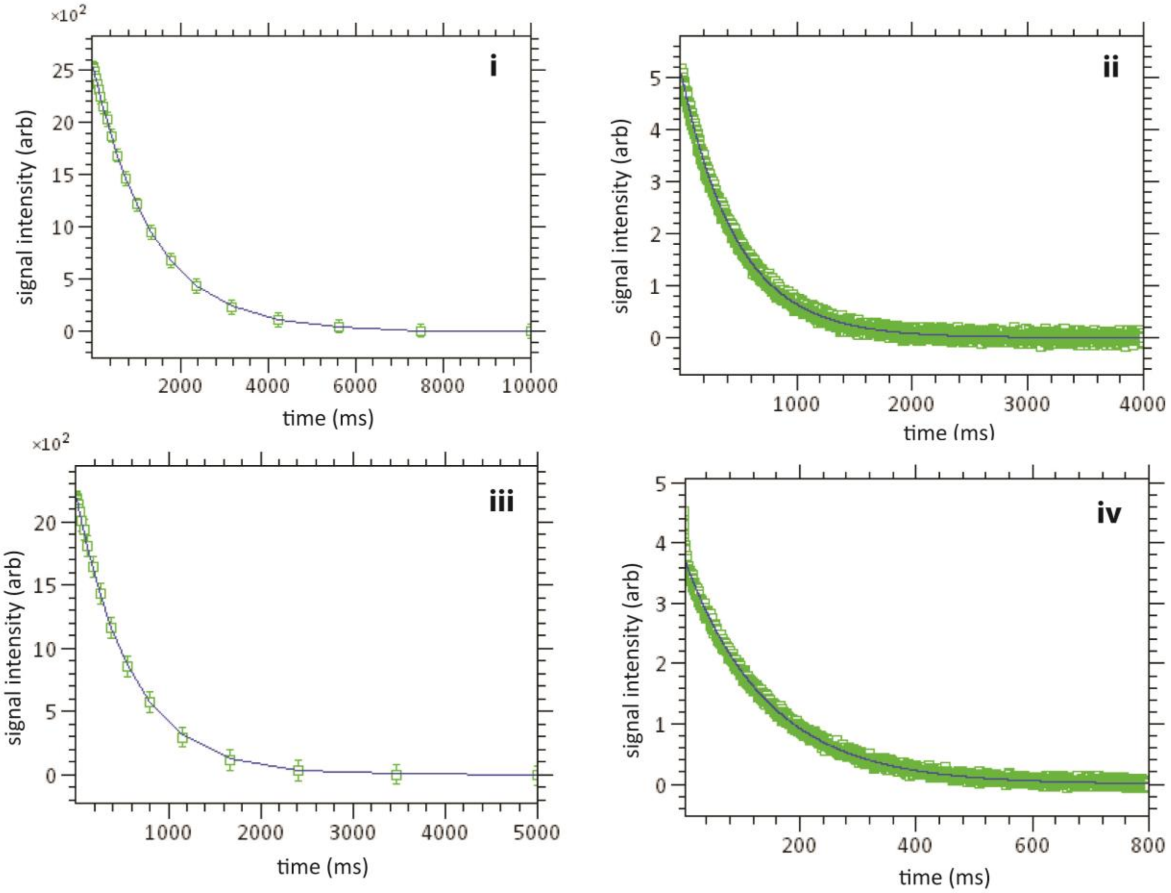

Supplementary Figure 10c: The raw decay signals of plasma as fitted by mono exponential curve. (i) T_1-relaxation, (ii) T_2-relaxation for plasma, and (iii) T_1-relaxation, (iv) T_2-relaxation for RBCs. The signal were collected using CPMG spin-echoes and inversion recovery (with modified CPMG observation) for T_2 relaxation, and T_1-relaxation measurement, respectively (Methods Online). The fitted values were (i) T_1= 1390 ms, (ii) T_2= 483 ms, (iii) T_1= 594 ms, and (iv) T_2= 142.3 ms.

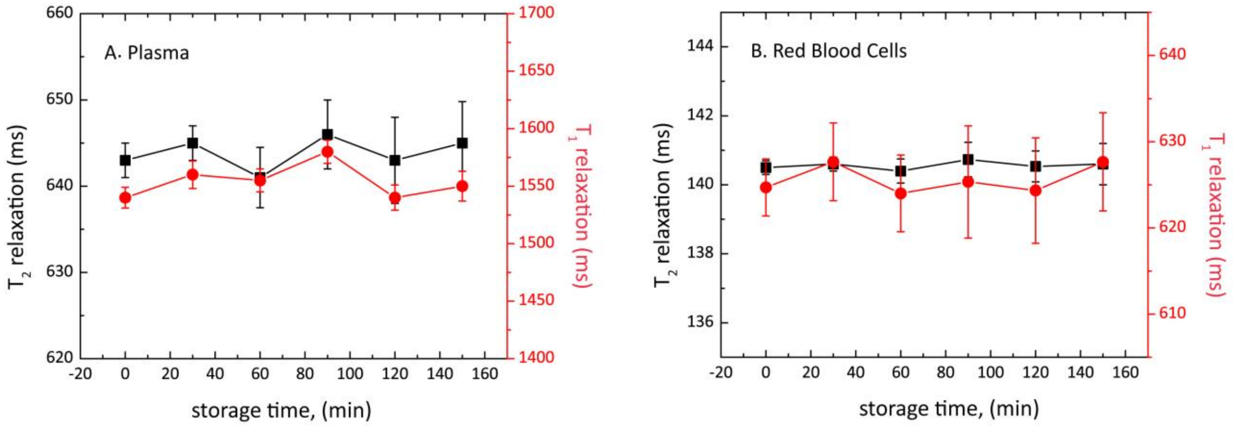

Supplementary Figure 10d: Sample stability as the function of time in 4^{0}C storage. (i) T_1-relaxation (left), (ii) T_2-relaxation (right) for (a) plasma, and (b) RBCs, respectively. The sample were brought to room temperature (26^{0}C) before micro MR analysis. For non-critical analysis, we used frozen down samples (up to a maximum of 3 days).

	**ESR**	**Mass Spec**	**UV-VIS**	**micro MR***
Marker	rare-spin nuclei	isoprostance	photo-sensitive molecules	proton nuclei
Principle	electron spin	mass	photo-absorption	relaxation properties
Sensitivity	low	high	mid	high
Specificity	high	high	mid to high	mid to high
Sample Preparation	tedious	tedious	easy	easy
Cost per test	low	high	ultra low	ultra low
Cost of instrumentation	mid to high	very high	low to mid	Low to mid
Point-of-care (size)	bench-top	lab-based	bench-top	bench-top
Rapid Test	No (hours)	No (hours)	Yes (minutes)	Yes (minutes)

Supplementary Table 1: State-of-the-art technological comparison. The advantageous and disadvantageous of the current state-of-art (*e.g.,* electron spin resonance (ESR), mass spectrometry (Mass Spec), UV-VIS spectrophotometry) against the work proposed here (micro MR)*.

	Urinary F_2-IsoP		NMR PoCT		Blood Glucose	
Subject no.	Obese	Lean	Obese	Lean	Obese	Lean
1	0.40	0.44	0.63	0.70	4.6	4.5
2	0.18	0.33	0.89	0.74	5.1	4.6
3	0.44	0.40	0.82	0.81	5.4	4.6
4	0.45	0.34	0.49	0.47	4.3	4
5	0.86	0.07	0.77	0.57	5	4.7
6	0.07	0.25	0.61	0.55	4.4	4.5
7	0.20	0.77	1.40	0.51	4.8	4.5
8	0.66	0.07	0.86	0.74	4.6	4.6
9	0.19	0.12	1.20	0.60	5.2	4.5
10	0.28	0.14	0.72	0.96	5.4	4.4
11	0.08	0.16	0.88	0.42	4.3	4.2
12	1.99	1.67	0.92	0.57	5.2	4.6
13	0.15	0.19	0.88	0.44	4.3	4.6
14	1.59	1.01	0.92	0.47	4.9	4.5
15	0.29	0.12	0.81	0.66	4.6	4.7
16	0.47	0.22	0.94	0.52	5.2	4.7
17	0.39	0.19	0.88	0.71	5.6	4.5
18	0.43	0.29	0.53	0.43	4.3	3.9
19	2.00	0.46	1.50	0.52	5.3	4.2
20	0.24	0.35	0.55	0.53	4.2	4.9
21	0.15	1.39	1.11	0.70	4.6	4.6

Supplementary Table 2: The oxidative stress in urine F_2-isoprostance/Cr (gold standard), the normalized oxidative stress in red blood cells (PoCT NMR), and fasting blood glucose taken from 21 anonymized subjects (Figure 8 in Main Text). The F_2-IsoP samples were processed with chromatography–mass spectrometry (details in Methods Online).

Supplementary References

1. Aime, S., Fasano, M., Paoletti, S., Arnelli, A. & Ascenzi, P. NMR Relaxometric Investigation on Human Methemoglobin and Fluoromethemoglobin. An Improved Quantitativein Vitro Assay of Human Methemoglobin. *Magnetic Resonance in Medicine* **33**, 827–831 (1995).
2. Grover, N. Principles of biochemistry (4th ed.). *Biochem. Mol. Biol. Educ.* **34**, 162–163 (2006).
3. Peisach, J., Blumberg, W. E. & Rachmilewitz, E. A. The demonstration of ferrihemochrome intermediates in heinz body formation following the reduction of oxyhemoglobin A by acetylphenylhydrazine. *Biochimica et Biophysica Acta (BBA) - Protein Structure* **393**, 404–418 (1975).
4. *Red Cell Membrane Transport in Health and Disease*. (Springer Berlin Heidelberg, 2003). doi:10.1007/978-3-662-05181-8.
5. Rifkind, J. M., Abugo, O., Levy, A. & Heim, J. [28] Detection, formation, and relevance of hemichromes and hemochromes. in *Methods in Enzymology* vol. 231 449–480 (Elsevier, 1994).
6. Longa, S. D. *et al.* Iron site structure of two irreversible hemichromes from human hemoglobin, untreated and oxidized to sulfoxide at MetD6(55) β. *Biochimica et Biophysica Acta (BBA) - Protein Structure and Molecular Enzymology* **1294**, 72–76 (1996).
7. Roche, M., Rondeau, P., Singh, N. R., Tarnus, E. & Bourdon, E. The antioxidant properties of serum albumin. *FEBS Letters* **582**, 1783–1787 (2008).
8. Giulivi, C., Hochstein, P. & Davies, K. J. A. Hydrogen peroxide production by red blood cells. *Free Radical Biology and Medicine* **16**, 123–129 (1994).
9. Rifkind, J. M., Zhang, L., Levy, A. & Manoharan, P. T. The Hypoxic Stress on Erythrocytes Associated with Superoxide Formation. *Free Radical Research Communications* **13**, 645–652 (1991).
10. Quinlan, G. J., Martin, G. S. & Evans, T. W. Albumin: Biochemical properties and therapeutic potential. *Hepatology* **41**, 1211–1219 (2005).
11. Song, Y.-Q. *et al.* T1–T2 Correlation Spectra Obtained Using a Fast Two-Dimensional Laplace Inversion. *Journal of Magnetic Resonance* **154**, 261–268 (2002).
12. Winterbourn, C. C. [26] Oxidative reactions of hemoglobin. in *Methods in Enzymology* vol. 186 265–272 (Elsevier, 1990).
