# Supplementary material for: Molecular phenotyping of oxidative stress in diabetes mellitus with point-of-care NMR system
Source: NPJ Aging Mech Dis. 2020 Oct 5;6:11. doi: 10.1038/s41514-020-00049-0 (PMC7536436; doi:10.1038/s41514-020-00049-0)
Supplement: Supplementary file 2 — reporting sum [file 41514_2020_49_MOESM2_ESM.pdf]

nature research

Corresponding author(s): NPJAMD-00268R

Last updated by author(s): Jul 22, 2020

Reporting Summary

Nature Research wishes to improve the reproducibility of the work that we publish. This form provides structure for consistency and transparency in reporting. For further information on Nature Research policies, see our Editorial Policies and the Editorial Policy Checklist.

Statistics

For all statistical analyses, confirm that the following items are present in the figure legend, table legend, main text, or Methods section.

n/a	Confirmed	
☐	☒	The exact sample size (n) for each experimental group/condition, given as a discrete number and unit of measurement
☐	☒	A statement on whether measurements were taken from distinct samples or whether the same sample was measured repeatedly
☐	☒	The statistical test(s) used AND whether they are one- or two-sided<br>*Only common tests should be described solely by name; describe more complex techniques in the Methods section.*
☐	☒	A description of all covariates tested
☐	☒	A description of any assumptions or corrections, such as tests of normality and adjustment for multiple comparisons
☐	☒	A full description of the statistical parameters including central tendency (e.g. means) or other basic estimates (e.g. regression coefficient) AND variation (e.g. standard deviation) or associated estimates of uncertainty (e.g. confidence intervals)
☐	☒	For null hypothesis testing, the test statistic (e.g. F, t, r) with confidence intervals, effect sizes, degrees of freedom and P value noted<br>*Give P values as exact values whenever suitable.*
☒	☐	For Bayesian analysis, information on the choice of priors and Markov chain Monte Carlo settings
☒	☐	For hierarchical and complex designs, identification of the appropriate level for tests and full reporting of outcomes
☒	☐	Estimates of effect sizes (e.g. Cohen's d, Pearson's r), indicating how they were calculated

Our web collection on statistics for biologists contains articles on many of the points above.

Software and code

Policy information about availability of computer code

Data collection	Microsoft Excel
Data analysis	Origin 9.0

For manuscripts utilizing custom algorithms or software that are central to the research but not yet described in published literature, software must be made available to editors and reviewers. We strongly encourage code deposition in a community repository (e.g. GitHub). See the Nature Research guidelines for submitting code & software for further information.

Data

Policy information about availability of data

All manuscripts must include a data availability statement. This statement should provide the following information, where applicable:

- Accession codes, unique identifiers, or web links for publicly available datasets
- A list of figures that have associated raw data
- A description of any restrictions on data availability

Data availability. The data that support the findings of this study are available from the corresponding author (weng.kung@inl.int) upon reasonable request.

Field-specific reporting

Life sciences study design

All studies must disclose on these points even when the disclosure is negative.

Sample size	We have a evaluated a total of more than 200 subjects. These are walk-in patients. The effect desire were observed and calculated using T-Test otherwise the n-size was set
Data exclusions	blood sample with integrity problem will be rejected.
Replication	the study was conducted in triplicate manner.
Randomization	the patient samples are determined by the randomization of walk-in patients, who satisfied the inclusion/exclusion criteria designed.
Blinding	Doubly blinded fashion. Sample collector and sample measurements were conducted by two different personnel. None of this have access or knowledge about the sample. The blind will only open during final write-up and discussion.

Reporting for specific materials, systems and methods

We require information from authors about some types of materials, experimental systems and methods used in many studies. Here, indicate whether each material, system or method listed is relevant to your study. If you are not sure if a list item applies to your research, read the appropriate section before selecting a response.

Materials & experimental systems

n/a	Involved in the study	
☒	☐	Antibodies
☒	☐	Eukaryotic cell lines
☒	☐	Palaeontology and archaeology
☒	☐	Animals and other organisms
☐	☒	Human research participants
☐	☒	Clinical data
☒	☐	Dual use research of concern

Methods

n/a	Involved in the study	
☒	☐	ChIP-seq
☒	☐	Flow cytometry
☒	☐	MRI-based neuroimaging

Human research participants

Policy information about studies involving human research participants

Population characteristics	Subjects without past history of diabetes mellitus (DM) and had normal oral glucose tolerance test according to the American Diabetes Association criteria (fasting glucose <5.6 mmol/L; two hour post oral glucose tolerance test glucose of <7.8 mmol/L) were recruited into this study following provision of informed consent. They were Chinese males aged between 21 and 40 years, with a body mass index below 23.5 kg/m2.
Recruitment	Subjects with Diabetes Mellitus; anonymities residual samples collected from DM patients at the outpatient clinic for measurement of glycated hemoglobin (HbA1c) as part of their clinical care, were included in this study.
Ethics oversight	Institutional Review Board of the National Healthcare Group.

Note that full information on the approval of the study protocol must also be provided in the manuscript.

Clinical data

Policy information about clinical studies

All manuscripts should comply with the ICMJEguidelines for publication of clinical research and a completedCONSORT checklist must be included with all submissions.

Clinical trial registration	This study received approval from the local Institutional Review Board of the National Healthcare Group.
Study protocol	No drug trial is conducted.
Data collection	This study received approval from the local Institutional Review Board of the National Healthcare Group. The EDTA-anticoagulated whole blood samples were collected using standard phlebotomy procedures. All blood samples were kept at ≤4°C within two hours of collection and were kept refrigerated until analysis.
Outcomes	Only blood collection and in vitro measurement of blood were carried out. There is no bearing on therapeutic decision.
